# The Influence of PARP, ATR, CHK1 Inhibitors on Premature Mitotic Entry and Genomic Instability in High-Grade Serous *BRCA^MUT^* and *BRCA^WT^* Ovarian Cancer Cells

**DOI:** 10.3390/cells11121889

**Published:** 2022-06-10

**Authors:** Patrycja Gralewska, Arkadiusz Gajek, Dorota Rybaczek, Agnieszka Marczak, Aneta Rogalska

**Affiliations:** 1 Department of Medical Biophysics, Institute of Biophysics, Faculty of Biology and Environmental Protection, University of Lodz, Pomorska 141/143, 90-236 Lodz, Poland; patrycja.gralewska@edu.uni.lodz.pl (P.G.); arkadiusz.gajek@biol.uni.lodz.pl (A.G.); agnieszka.marczak@biol.uni.lodz.pl (A.M.); 2 Department of Cytophysiology, Faculty of Biology and Environmental Protection, University of Lodz, Pomorska 141/143, 90-236 Lodz, Poland; dorota.rybaczek@biol.uni.lodz.pl

**Keywords:** ATR inhibitor, CHK1 inhibitor, ovarian cancer, PARP inhibitor, replication stress, targeted therapy

## Abstract

Olaparib is a poly (ADP-ribose) polymerase inhibitor (PARPi) that inhibits PARP1/2, leading to replication-induced DNA damage that requires homologous recombination repair. Olaparib is often insufficient to treat *BRCA*-mutated (*BRCA^MUT^*) and *BRCA* wild-type (*BRCA^WT^*) high-grade serous ovarian carcinomas (HGSOCs). We examined the short-term (up to 48 h) efficacy of PARPi treatment on a DNA damage response pathway mediated by ATR and CHK1 kinases in *BRCA^MUT^* (PEO-1) and *BRCA^WT^* (SKOV-3 and OV-90) cells. The combination of ATRi/CHK1i with PARPi was not more cytotoxic than ATR and CHK1 monotherapy. The combination of olaparib with inhibitors of the ATR/CHK1 pathway generated chromosomal abnormalities, independent on *BRCA^MUT^* status of cells and formed of micronuclei (MN). However, the beneficial effect of the PARPi:ATRi combination on MN was seen only in the PEO1 *BRCA^MUT^* line. Monotherapy with ATR/CHK1 inhibitors reduced BrdU incorporation due to a slower rate of DNA synthesis, which resulted from elevated levels of replication stress, while simultaneous blockade of PARP and ATR caused beneficial effects only in OV-90 cells. Inhibition of ATR/CHK1 increased the formation of double-strand breaks as measured by increased γH2AX expression at collapsed replication forks, resulting in increased levels of apoptosis. Our findings indicate that ATR and CHK1 inhibitors provoke premature mitotic entry, leading to genomic instability and ultimately cell death.

## 1. Introduction

Ovarian cancer is the leading cause of death from gynecological malignancies [1]. High-grade serous ovarian carcinoma (HGSOC) accounts for approximately 50% of ovarian cancers and is characterized by *TP53* mutations (96%), homologous recombination (HR) DNA repair defects (50%), cyclin E1 (CCNE1) amplification and genomic instability [2]. The cells often carry *BRCA1* or *BRCA2* (*BRCA1/2*) mutations, germline or somatic mutations in ataxia telangiectasia and Rad3-related protein (ATR) or checkpoint kinase 1 (CHK1), as well as mutations in genes associated with the HR pathway [3]. Due to *TP53* mutations, HGSOCs lose their G1 checkpoint control, resulting in increased dependence on S and G2 checkpoints [4].

It is estimated that about 10,000 single-strand breaks (SSBs) of DNA occur daily in a mammalian cell. DNA damage induced by olaparib treatment has been shown to significantly increase genetic instability [5]. Olaparib and veliparib, poly (ADP-ribose) polymerase inhibitors (PARPi), induce genomic instability, resulting in a marked increase in sister chromatid exchange (SCE) frequencies and chromatid-type aberrations. Olaparib induces chromatid-type aberrations, including gaps, breaks, radial chromosomes and telomere associations [6]. Genomic instability in olaparib-treated cells manifests as the extensive accumulation of gaps, breaks, radial chromosomes, and micronuclei (MN) in response to aberrant double strand break (DSB) repair [7]. In addition, PARP1 inhibition in cancer cells may lead to the upregulation of the HR repair pathway to maintain cell viability [8]. Although PARPi can delay cancer progression, they do not improve overall survival [9]; thus, there is an urgent need to develop new, more successful therapies. One effective strategy to potentiate PARP cytotoxicity in ovarian cancer cells would be to target cell cycle checkpoints and force mitotic entry with DSBs, thereby inducing cell death. 

Our previous study confirmed that the inactivation of cell cycle checkpoint kinases such as ATR or CHK1 may increase the cytotoxicity of PARPi after 5 days of treatment [10]. In response to DNA damage, ATR phosphorylates CHK1 protein, which in turn mediates CDC25A-C phosphorylation, leading to the inhibition of CDK1 and CDK2 (thus preventing cell cycle progression) [11,12]. DSB lesions lead to the activation of the G2/M cell cycle checkpoint, which blocks entry into mitosis [13]. Progression through mitosis has previously been shown to promote PARPi cytotoxicity in HR-deficient cells [14].

The effect of ATR and CHK1 kinases in response to PARPi treatment on cell cycle disorders and its consequences in ovarian cancer cells remain unclear. Genetic predisposition (*BRCA1/2* mutations) or phenotypic characteristics (platinum resistance) are not sufficient to predict patient response to olaparib treatment [15]. Whether DNA damage from replication stress (RS) produces an efficient G2/M checkpoint response in ovarian cancer is not known. Thus, in this study, we investigated the role of ATR and CHK1 inhibition at the early stage of olaparib response in *BRCA2^MUT^* (PEO-1) and *BRCA^WT^* (OV-90 and SKOV-3) ovarian cancer cells and sought to further elucidate the mechanisms leading to ovarian cancer cell death. Here, we report that PARPi has a cytostatic effect, while its cytotoxic effects and diminished DNA repair are caused by ATRi and CHK1i monotherapy. The combination of olaparib with inhibitors of the ATR/CHK1 pathway generated chromosomal abnormalities, independent on *BRCA^MUT^* status of cells and formed of micronuclei. ATR and CHK1 inhibitors provoke premature mitotic entry and provide genomic instability to ovarian cancer cells.

## 2. Materials and Methods

### 2.1. Reagents

Culture media (RPMI 1640, DMEM) and fetal bovine serum (FBS) were obtained from Gibco (Thermo Fisher Scientific, Waltham, MA, USA). Trypsin-EDTA was acquired from Sigma-Aldrich (St. Louis, MO, USA). Violet Chromatin Condensation/Dead Cell Apoptosis Kit (Cat no: A35135) and CLICK EDU (Cat no: C10337) were from Thermo Fisher Scientific. The FITC BrdU Flow Kit was purchased from BD Biosciences (Franklin Lakes, NJ, USA). PARPi (AZD2281), ATRi (AZD6738), and CHK1i (MK8776) were purchased from Selleckchem (Houston, TX, USA). Other chemicals and solvents were of high analytical grade and were obtained from Sigma-Aldrich or Avantor Performance Materials Poland S.A. (Gliwice, Poland).

### 2.2. Cell Culture and Drug Administration

The human OV-90 cell line with mutated *TP53* gene (human malignant papillary serous carcinoma, American Type Culture Collection (ATCC) CRL-11732™) and SKOV-3 cell line with loss of *TP53* function (*TP53* null) (human ovarian adenocarcinoma, ATCC HTB-77) were purchased from ATCC (Rockville, MD, USA). *BRCA^MUT^* PEO-1 cells (human ovarian cancer; estrogen rec, 10032308) were obtained from the European Collection of Authenticated Cell Cultures. The newly acquired cells were expanded, and aliquots of less than 10 passages were stored in liquid nitrogen. All cell lines were kept at a low passage, returning to original frozen stocks every 6 months. During the course of the study, cells were thawed and passaged within 2 months of each experiment. Cells were cultured in DMEM and RPMI with 10% FBS and regularly checked for mycoplasma contamination. Cells were cultured in an atmosphere of 5% CO_2_ and 95% air at 37 °C.

### 2.3. MTT Assay

Logarithmically growing cells (1 × 10^4^) were seeded into 96-well plates and treated with the indicated doses of PARPi (AZD2281), CHK1i (MK8776), and ATRi (AZD6738) for 24 and 48 h. After treatment, cells were washed twice with PBS, and incubated with 50 µL MTT (at a final concentration of 0.5 mg/mL) (Sigma Aldrich, St. Louis, MO, USA) for 4 h. The medium in each well was aspirated and violet formazan crystals that formed as a result of MTT reduction within metabolically viable cells were dissolved in 100 µL DMSO per well. Absorbance was measured at 570 nm with a microplate reader (Awareness Technology Inc., Palm City, FL, USA) [16]. To analyze the drug interactions between olaparib combined with ATRi and CHK1i, the coefficient of drug interaction (CDI) was calculated as described previously [17].

### 2.4. Metaphase Spread

Cells were treated with the inhibitors for 24 h, then harvested for chromosome preparations using colcemid (50 ng/mL) for 90 min, followed by incubation with 0.075 mol/L potassium chloride (KCl) for 18 min at 37 °C. Following drop wise addition of Carnoy fixative (3:1 methanol:acetic acid), samples were incubated in the fixative for one hour, pelleted at 1000 g, and incubated in fresh fixative at 4 °C overnight. After replenishing the fixative again, the fixed cells were placed onto uncoated microscope slides and dried for at least 24 h at room temperature. Slides were stained in Giemsa staining solution (Aqua-med, Łódź, Poland) for 4 min, then analyzed for total gaps and breaks using a 100× objective and a Nikon ECLIPSE E600W (Nikon, Warsaw, Poland) fluorescent microscope. Fifty metaphases were scored for each sample in two independent experiments. An index of aberrations (M-phase aberrant cells) was calculated as the percent ratio between the number of cells showing chromosomal aberrations and all mitotic cells. Quantification of the number of aberrant M-phase cells (AI: aberration index) and nuclear phenotypes in cells was determined by counterstaining with Giemsa and DAPI (0.1 mg/mL; 4′,6-diamidino-2-phenylindole; Sigma Aldrich, Saint Quentin, France) for 5 min at room temperature. PEO-1, OV-90 and SKOV-3 cells were observed using an AxioImagerA1 fluorescence microscope (ZEISS, Jena, Germany) equipped with an UV-2A filter (UV-light; λ = 518 nm). All images were recorded at exactly the same time of integration with an AxioCam MRc5 CCD camera (ZEISS, Jena, Germany).

### 2.5. Micronucleus Assay

Cells (2 × 106) were seeded onto 100 mm Petri dishes and cultured for 24 h before treatment with olaparib, ATRi, CHK1i, and a combination of PARPi with ATRi or PARPi with CHK1i, each at a concentration of 4 µM. After 48 h of exposure, cells were washed twice with PBS, collected and incubated in a chilled hypotonic solution of KCl (75 mM) for 30 min. Samples were then centrifuged at 200× *g* for 5 min at 25 °C and fixed with Carnoy’s fixative (3:1 ratio of methanol:glacial acetic acid) for 5 min. After centrifugation at 200× *g* for 5 min, cells were resuspended in fresh Carnoy’s fixative. This step was repeated twice. Finally, cells were placed on microscopic slides, fixed with methanol for 15 min, air dried and stained with acridine orange for 2 min (10 μg/mL in PBS). Micronuclei (MN) were examined under an Optiphot-2 fluorescence microscope (Nikon) equipped with a B-2A filter (blue light; λ = 495 nm). The MN images presented were visualized by fluorescence microscopy (Olympus IX70, Tokyo, Japan; magnification 400×).

### 2.6. Determination of Proliferation Rate

To determine the OV-90, SKOV-3 and PEO-1 cell proliferation rates, we employed the trypan blue exclusion method [18]. Cells, seeded at a density of 2 × 105, were treated with the drugs and counted 24–168 h after treatment. Briefly, 4% trypan blue solution was mixed with the cell suspension in a ratio of 1:1, transferred to a Thoma chamber and viable/nonviable cells were counted under an optical microscope. Based on the number of cells at the beginning and at each studied time point, we calculated the doubling time using the following formula td = t/log2 (Nt/N0), where td is the time required for duplication of cell number, t is the time interval between the initial and final calculation of cell number, and N0 and Nt are the number of cells at the beginning and end of the experiment, respectively [19,20].

### 2.7. Cell-Cycle Analysis

Cell cycle distribution was quantified using a FITC-BrdU Flow Kit (BD Biosciences, San Jose, CA, USA) according to the manufacturer’s protocol. Briefly, cells (1 × 106) were plated onto 100 mm Petri dishes and once attached were treated with drugs for 24 h. Ten μM of bromodeoxyuridine (BrdU) was added to the culture medium and incubated for 2 h. Cells were harvested, fixed and incubated with FITC-conjugated anti-BrdU and 7-AAD solution for 15 min at room temperature, then analyzed immediately in a flow cytometer (LSR II, Becton Dickinson, San Jose, CA, USA). The mitotic index was calculated as the percent ratio between the number of dividing cells and the entire cell population by labeling cells with FITC-conjugated anti-BrdU and 7-AAD. The population of cells at specific phases of the cell cycle were quantified from a standard count of 10,000 cells using FlowJo software v7.6 (Ashland, OR, USA).

### 2.8. Immunofluorescence and Immunocytochemistry Staining

Cells (1.5 × 104 cells/well) were cultured for 24 h on ten-chambered glass slides (Greiner Bio-One, Frickenhausen, Germany), then treated with the compounds for 48 h. Next, the cells were fixed with paraformaldehyde (4% *w*/*v* in PBS), incubated with blocking buffer for 60 min (PBS/5% normal goat serum/0.3% Triton X-100), followed by incubation with primary antibodies against caspase-3 (cat.: 9664, at 1:400) and phospho-histone H2AX (Ser139) (cat.: 9718, at 1:400) prepared in Antibody Dilution Buffer (PB/1% BSA/0.3% Triton X-100). The secondary antibodies used were anti-rabbit IgG (H+L), F(ab’)2 fragment (Alexa Fluor 555 conjugate, at 1:1000) to detect caspase-3, and anti-rabbit IgG (H+L), F(ab’)2 fragment (Alexa Fluor 488 conjugate, at 1:2000) to detect phospho-histone H2AX. Nuclei were stained with DAPI (Vectashield mounting medium, cat no.: H-1200, Vector Laboratories, Newark, CA, USA) and 3,3′-dihexyloxacarbocyanine iodide (DiOC6, cat no: 318426, Sigma Aldrich, Steinheim am Albuch, Germany; 1 μM) was used to visualize cells with caspase-3. For visualization of membranes stained with DiOC6, a supercontinuum laser with 485 nm excitation and emission at 538–595 nm was applied. For nuclei stained with DAPI, the excitation and emission parameters used were 405 nm and 460–480 nm, respectively. Immunofluorescence images were acquired using a confocal laser scanning microscope (SP8, Leica Microsystems AG, Wetzlar, Germany) equipped with a 63× oil immersion objective [21].

### 2.9. Measurement of Chromatin Condensation

The Violet Chromatin Condensation/Dead Cell Apoptosis Kit with Vybrant^®^ DyeCycle™ Violet and SYTOX^®^ AADvanced™ was used to examine chromatin condensation during apoptosis (cat. no. A35135; Molecular probes^®^, Invitrogen™, Waltham, MA, USA). Briefly, 1 × 106 control and drug-treated cells were washed and resuspended in 1 mL Hank’s Balanced Salt Solution buffer (HBSS) containing Vybrant^®^ DyeCycle™ Violet and SYTOX^®^ AADvanced™ dyes, according to the manufacturer’s protocol. After 30 min of incubation (protected from light), stained cells were analyzed immediately without washing by flow cytometry (LSR II, Becton Dickinson, Franklin Lakes, NJ, USA) using ~405/488 nm dual excitation, while measuring the fluorescence emission at ~440/660 nm [22].

### 2.10. Detection of S-Phase Progression Using 5-Ethynyl-2′-deoxyuridine(EdU) Incorporation with Click-iT Chemistry

Cells were labeled for 30 min with 100 μM EdU (Life Technologies, Invitrogen, Paisley, Renfrewshire, UK), then fixed in 4% (*w/v*) paraformaldehyde (Merc, St. Louis, MO, USA) for 45 min and permeabilized with 1% Triton X-100 (Merc, St. Louis, MO, USA) for 20 min. For EdU staining, the Click-iT Alexa Fluor 488 Imaging Kit was used according to the manufacturer’s instructions, with some minor modifications. Briefly, cells were rinsed twice with 1% bovine serum albumin (BSA; Merc, St. Louis, MO, USA) and incubated for 30 min at 20 °C with 250 μL EdU Click-iT reaction cocktail per well. After removing the reaction cocktail, each well was washed once with 1% BSA. Cells were visualized using a fluorescence microscope (Zeiss, Jena, Germany) equipped with GFP and DAPI filters. Analysis was performed with AxioVision software (Zeiss, Jena, Germany). All images were recorded at exactly the same exposure time on an AxioCam MRc5 CCD camera (Zeiss, Jena, Germany).

### 2.11. Measurement of Phosphatidylserine Externalization

Double staining of cells with annexin V and propidium iodide was used to assess the first stage of apoptosis. This method is a useful tool for distinguishing viable cells (unstained with either fluorochrome) from apoptotic cells (stained with annexin V) and necrotic (dead) cells (stained with PI). Visualization of cells stained with annexin V- Alexa Fluor™ 488 and PI was applied according to the protocol of the manufacturer (cat: V13245, Invitrogen, Thermo Fisher Scientific, Waltham, MA, USA). Briefly, 4 × 10^5^ control and drug treated cells after 24 and 48 h were washed with cold PBS and resuspended in 140 μL binding buffer (delivered from producer) that contained 3 μL of annexin V-Alexa Fluor™ 488, 1 μL 100 μg/mL of PI and stained for 15 min on ice. Finally, at least 10^4^ cells were analyzed for Alexa Fluor™ 488 and PI fluorescence (Ex ~488 nm; Em ~530 nm) using a flow cytometer (LSR II, Becton Dickinson, Franklin Lakes, NJ, USA). With the use of the annexin V- Alexa Fluor™ 488 and propidium iodide (PI) double staining regime, three populations of cells were distinguishable in a two color flow cytometry: normal cells: annexin V- Alexa Fluor™ 488 negative, PI negative; apoptotic cells: annexin V-Alexa Fluor™ 488 positive, PI negative; dead cells: annexin V-Alexa Fluor™ 488 positive and PI positive; annexin V-Alexa Fluor™ 488 negative and PI positive. The populations of cells were quantified from a standard count of 10^4^ cells using FlowJo software v7.6 (Ashland, OR, USA).

### 2.12. Morphological Assessment of Apoptosis and Necrosis: Double Staining with Hoechst 33258 and Propidium Iodide (PI)

To determine the ratio between live, apoptotic, and necrotic cellular fractions, simultaneous cell staining with Hoechst 33258 and PI was performed as described previously [23]. These fluorescent dyes vary in their spectral characteristics and ability to penetrate cells. Analysis was performed with the Olympus IX70 fluorescence microscope. Cells were cultured with the drugs for 48 h, trypsinized, centrifuged, and resuspended in PBS to a final concentration of 1 × 10^6^ cells/mL. One µL of Hoechst 33258 (0.13 mM) and 1 µL of PI (0.23 mM) were added to 100 µL of the cell suspension. At least 100 cells were counted under a microscope on each slide and each experiment was performed in triplicate. Cells were classified as live, apoptotic or necrotic on the basis of their morphological and staining characteristics, and the percentage of specific cellular fractions was determined from the total number of cells. Cells were classified as live (bright blue fluorescence), early apoptotic cells (cells showing intensive blue fluorescence), late apoptotic cells (blue–violet stained cells with concomitant apoptotic morphology), and necrotic cells (red fluorescence) [24].

### 2.13. Western Blot Analysis

Drug-treated cells were lysed in cell extraction buffer (Invitrogen™) containing a protease inhibitor cocktail and PMSF (Sigma-Aldrich) in accordance with the manufacturer’s protocol. The protein concentration was determined using the Bradford method. Proteins (70 µg per lane) were separated by SDS polyacrylamide gel electrophoresis and transferred onto 0.45 µm PVDF membranes using semi-dry transfer with the Trans-Blot Turbo Transfer System (Bio-Rad). After blocking nonspecific sites with 5% non-fat dry milk, membranes were incubated with rabbit monoclonal antibodies (1/1000) against caspase-3 (cat no.: #9664) and phospho-histone H2AX (Ser139) (cat.no.: #9718) from Cell Signaling Technology, Inc. (Danvers, MA, USA), and mouse monoclonal anti-β-actin antibodies (cat. A1878, Sigma-Aldrich). Membranes were then incubated with anti-rabbit IgG horseradish peroxidase-conjugated (cat.: 7074, Cell Signaling Technology) or anti-mouse IgG HRP-conjugated (cat: A28177, Invitrogen, Thermo Fisher Scientific, Waltham, MA, USA) secondary antibodies, followed by incubation with a chemiluminescent substrate (SuperSignal™ West Pico PLUS Chemiluminescent Substrate or SuperSignal™ West Atto Ultimate Sensitivity Substrate, Thermo Fisher Scientific, Waltham, MA, USA). Immunoreactive bands were visualized using a c300 imaging system (Azure Biosystems, Dublin, CA, USA). Band intensities were quantified using ImageJ software v1.5 (NIH, Bethesda, MD, USA). The integrated optical density of the bands was measured in a digitized image. Relative protein levels were expressed as the ratio of the densitometric volume of the tested band to that of the respective β-actin band [10].

### 2.14. Statistical Analysis

Data are presented as the mean ± SD of at least three independent experiments. Statistical analyses were performed with the Student’s *t*-test and one-way ANOVA, with the Tukey post hoc test for multiple comparisons, as appropriate (StatSoft, Tulsa, OK, USA). *p*-values of <0.05 were considered statistically significant.

## 3. Results

### 3.1. CHK1 or ATR Inhibition Is Cytotoxic to Ovarian Cancer Cells

In our previous study, we demonstrated that using PARPi:ATRi and PARPi:CHK1i at a concentration ratio of 1:1 was the most effective combination [10]. Thus, in the present study, cells were incubated with the test compounds at a ratio of 1:1 for 24 and 48 h at a concentration of 4 µM.

**Figure 1:**
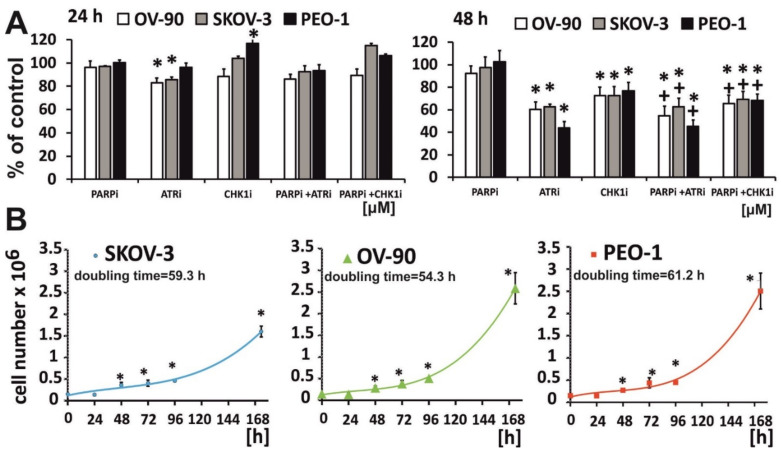
CHK1i or ATRi decreased cell (**A**) Cell viability after treatment with PARPi, CHK1i, and ATRi in *BRCA^MUT^* (PEO-1) and *BRCA^WT^* (OV-90, SKOV-3) cells at a concentration of 4 µM for 24 and 48 h was assessed by the MTT assay; (**B**) Cell doubling time was calculated from the cell growth curve during the exponential growth phase using the formula, Td = t/log2 (Nt/N0). * indicates statistically significant differences between samples incubated with the compound compared with control cells (*p* < 0.05); + indicates statistically significant differences between samples incubated with PARPi alone and the combination treatments (PARPi:ATRi; PARPi:CHK1i) (*p* < 0.05).

The tested combinations, as well as olaparib or CHK1i monotherapy, did not significantly affect the cell survival rate after 24 h (Figure 1A). After 48 h of incubation, ATRi was more cytotoxic in the *BRCA^MUT^* cell line (PEO-1), reducing the cell viability to 44% compared to approximately 60% in the *BRCA^WT^* cell lines (SKOV-3 and OV-90). In contrast, CHK1i monotherapy was slightly more cytotoxic in the *BRCA^WT^* cell lines (72%) compared to the *BRCA^MUT^* cells (PEO-1, 77% viable cells). PARPi:ATRi combination treatment was the most cytotoxic in PEO-1 cells, decreasing cell viability to 45% compared to 61% in OV-90 and SKOV-3 cells. The combinations treatment did not significantly affect the cytotoxicity compared to ATRi or CHK1i monotherapy, although it was more cytotoxic than PARPi treatment. Treatment with the PARPi:CHK1i combination was less cytotoxic than PARPi:ATRi, reducing the cell viability to 68% in ovarian cancer cells; however, the beneficial effect of PARPi:CHK1i combination at the selected concentrations after 48 h was more visible, as calculated by the coefficient of drug interaction (CDI) (PARPi:CHK1i: SKOV-3, CDI = 0.97; OV-90, CDI = 0.97; PEO-1, CDI = 0.87). As shown in the growth curves in Figure 1B, the average doubling time of the untreated cells was 54.3 h in OV-90 cells, 57.6 h in SKOV-3 cells and 59.9 h in PEO-1 cells.

### 3.2. ATRi and CHK1imonotherapy Increases Apoptosis and DNA Damage

**Figure 2:**
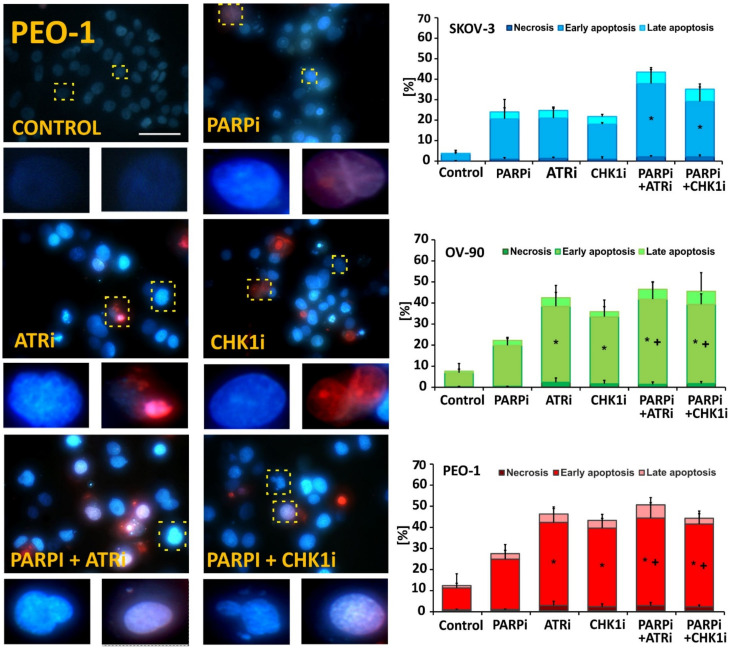
ATRi/CHK1i monotherapy and combination treatment caused cell death. Representative fluorescence images of the apoptotic and necrotic changes caused by the compounds in PEO-1 cells. Representative images in SKOV-3 and OV-90 cells are shown in Supplementary Figure S1. Apoptotic and necrotic changes were visualized after double staining with Hoechst 33258/PI under a fluorescence microscope (Olympus IX70; scale bar 50 µm, magnification 400×). Representative cells are marked with a dashed line and enlarged. The cells were divided into four categories as follows: live cells (dark blue fluorescence), early apoptotic (bright blue fluorescence), late-apoptotic (pink–violet fluorescence), and necrotic cells (red fluorescence). Data are from three independent assays and are presented as the mean ± SD. * indicates statistically significant differences between treated samples and control cells (*p* < 0.05); + indicates statistically significant differences between samples incubated with PARPi alone and the combination treatments (PARPi:ATRi; PARPi:CHK1i) (*p* < 0.05).

Morphological changes induced by the inhibitors were assessed by Hoechst 33258/PI double staining (Figure 2 and Supplementary Figure S1). The greatest apoptotic changes were observed in the *BRCA^MUT^* cell line (PEO-1). Olaparib monotherapy did not significantly increase the frequency of apoptotic cells, whereas ATRi monotherapy was the most potent in terms of apoptosis induction, leading to increases in the frequency of early apoptotic cells of 40% in PEO-1 cells and 36% in OV-90 cells, but only 19% in SKOV-3 cells. Combination treatment of PARPi with ATRi caused an almost 2-fold increase in early and late apoptotic cellular fractions compared to treatment with PARPi alone in all tested cell lines (PARPi:ATRi: SKOV-3, 40%; OV-90, 45%; PEO-1, 48% compared to PARPi alone: SKOV-3, 22%; OV-90, 22%; PEO-1, 27%) (Figure 2). PARPi:CHK1i combination treatment also led to significant changes in the apoptotic cellular fractions compared to PARPi treatment in OV-90 and PEO-1 cell lines. Nevertheless, no differences were observed between the performance of the combination treatment compared with ATRi or CHK1i.

**Figure 3:**
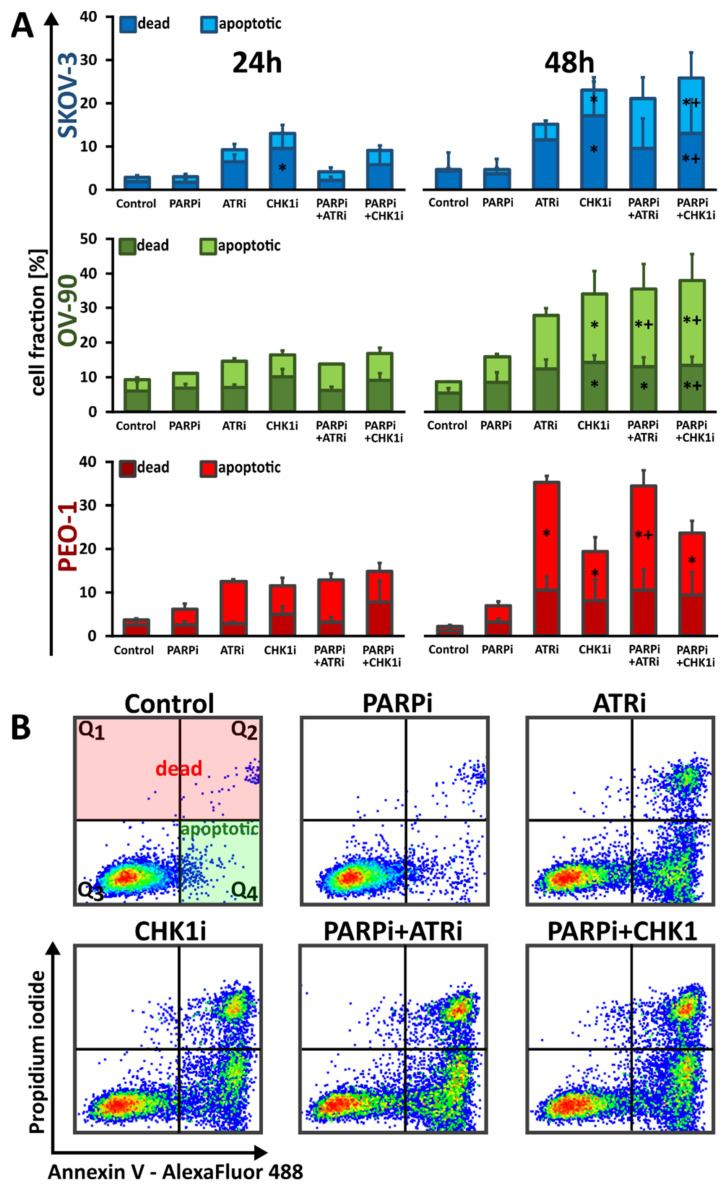
ATRi/CHK1i monotherapy and combination treatment caused phosphatidylserine externalization. (**A**) Percentage of apoptotic and dead SKOV-3, PEO-1 and OV-90 cells after 24 and 48 h treatment with PARPi (4 µM), ATRi (4 µM) or CHK1i (4 µM) alone or in combination. Data are presented as mean ± SD of 3 independent experiments. * indicates statistically significant differences between samples incubated with the compound compared with control cells (*p* < 0.05); + indicates statistically significant differences between samples incubated with PARPi alone and the combination treatments (PARPi:ATRi; PARPi:CHK1i) (*p* < 0.05). (**B**) Representative dot plots showing induction of apoptosis and fraction of dead PEO-1 cells after 48 h treatment with PARPi (4 µM), ATRi (4 µM) or CHK1i (4 µM) alone and in combination. Data for SKOV-3 and OV-90 cells are shown in Supplementary Figure S2. Individual samples are presented as data points. The population of apoptotic cells was calculated according to the presented gating strategy.

The exposure of phospholipid phosphatidylserine (PS) on the cell membrane represents a major characteristic of the caspase-dependent apoptosis process. Thus, annexin V, which is a PS-binding protein, is used to detect PS externalization. In order to distinguish between apoptotic and nonapoptotic cells, annexin V was combined with cell impermeable dye, propidium iodide (PI). Olaparib monotherapy was not sufficient to induce apoptosis within the tested time range and concentration (Figure 3 and Supplementary Figure S2). ATRi monotherapy effect was the most prominent in PEO-1 cells, where after 48 h of drug treatment the apoptotic cell fraction equaled 25%. CHK1i monotherapy was the most effective in OV-90 cell line, where the apoptotic cell fraction reached 20%. PARPi:ATRi combination increased apoptosis in OV-90 and PEO-1; however, PARPi:ATRi mediated apoptotic changes were the most visible in PEO-1 cells compared to olaparib monotherapy. PARPi:CHK1i combination effect was more pronounced in SKOV-3 and OV-90 cell lines. Despite the upward trends, no statistically significant differences were noted between the effect of the combination of PARPi:ATRi and PARPi:CHK1i compared to monotherapy with ATRi or CHK1i in the studied time range.

**Figure 4:**
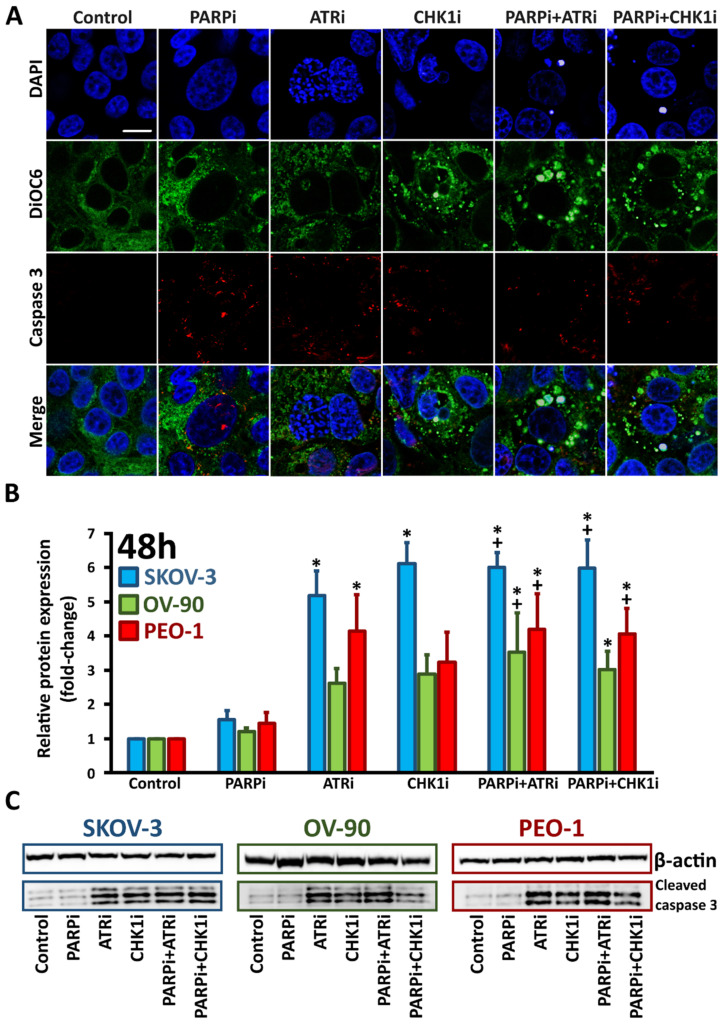
ATRi/CHK1i treatment increases caspase-3 expression. (**A**) For immunofluorescence staining, PEO-1 cells were incubated with PARPi, ATRi or CHK1i alone, or the combination of PARPi:ATRi or PARPi:CHK1i at 4 µM for 48 h and labeled with antibodies against caspase-3 (red colour). Representative images for SKOV-3 and OV-90 cell lines are shown in Supplementary Figure S3. Images were acquired using a confocal laser scanning microscope (scale bar 20 µm, magnification 63×); (**B**) Relative expression of cleaved caspase-3 in the cells. * indicates statistically significant differences between samples incubated with the compound compared with control cells (*p* < 0.05); + indicates statistically significant differences between samples incubated with PARPi alone and the combination treatments (PARPi:ATRi; PARPi:CHK1i) (*p* < 0.05); (**C**) Representative Western blot images.

Caspases are key mediators of DNA fragmentation. Caspase-3, a protein activated in both extrinsic and intrinsic apoptosis pathways, was used as a marker of apoptosis. We found that cleaved caspase-3 expression, as well as the accumulation of the DNA damage marker γH2AX, was not significantly increased following PARPi monotherapy in *BRCA^MUT^* and HR-proficient cells. Treatment with ATRi or CHK1i increased cleaved caspase-3 levels (SKOV-3, 5.2-fold (ATRi) and 6.12-fold (CHK1i); OV-90, 2.6-fold (ATRi) and 2.9-fold (CHKi); PEO-1, 4.2-fold (ATRi) and 3.2-fold (CHKi)) compared to control cells (Figure 4B). Each combination treatment was found to be more effective than treatment with PARPi alone. Although cleaved caspase-3 expression levels were not significantly increased in combination treatments compared to ATRi or CHK1i monotherapy, we did find that compared to PARPi treatment alone, cleaved caspase-3 levels were increased 6-fold, 3.5-fold and 4.2-fold in SKOV-3, OV-90 and PEO-1 cells, respectively, after PARPi:ATRi and PARPi:CHK1i combination treatments.

An increase in γH2AX expression was observed after treatment with ATRi or CHK1i (SKOV-3, 28.8-fold (ATRi) and 44.1-fold (CHKi); OV-90, 4.9-fold (ATRi) and 5.5-fold (CHKi); PEO-1, 2.1-fold (ATRi) and 1.8-fold (CHKi)). PARPi in combination with CHK1i increased γH2AX phosphorylation compared with PARPi alone by 32.2-fold in SKOV-3 cells, 6.3-fold in OV-90 cells and 2.1-fold in PEO-1 cells. Similarly, PARPi treatment in combination with ATRi increased γH2AX expression compared with PARPi alone by 28.4-fold in SKOV-3 cells, 5.6-fold in OV-90 cells and 2.2-fold in PEO-1 cells. The combination treatment did not significantly increase γH2AX expression compared to ATRi or CHK1i monotherapy. Although the largest changes in protein expression levels were found in *BRCA^WT^* SKOV-3 cells, this cell line was found to have the lowest protein expression levels in the control cells (Supplementary Figure S5).

**Figure 5:**
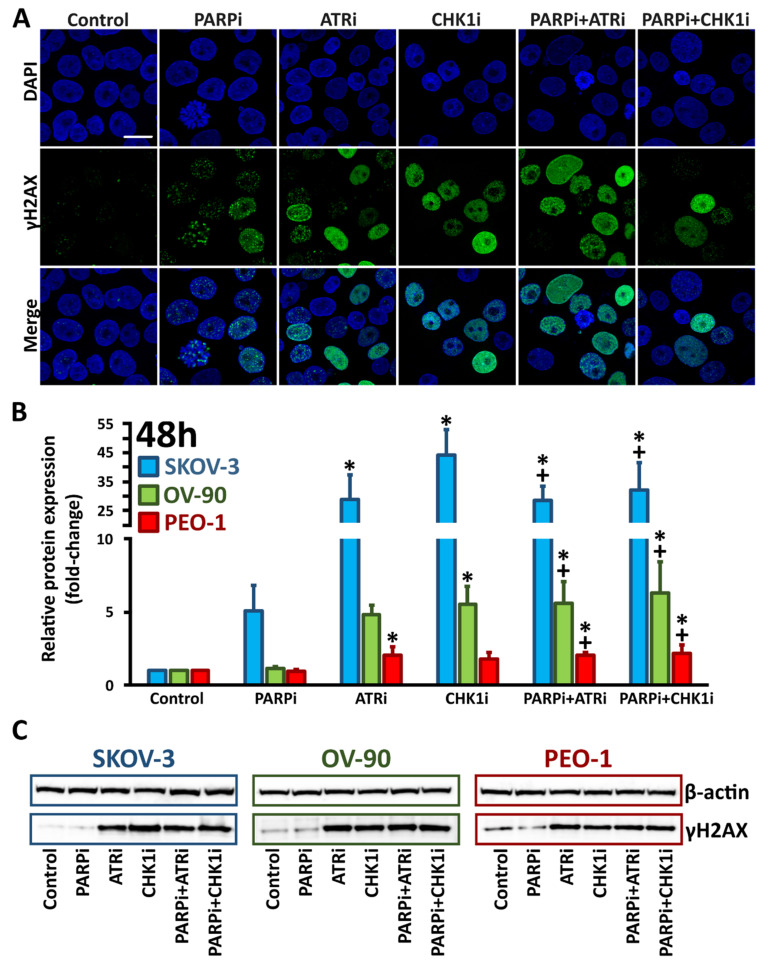
ATRi/CHK1i increases γH2AX expression. (**A**) For immunofluorescence staining, PEO-1 cells were treated with PARPi, ATRi or CHK1i, or a combination of PARPi:ATRi or PARPi:CHK1i at 4 µM for 48 h and labeled with antibodies against γH2AX (green colour). Representative images in SKOV-3 and OV-90 cells are shown in Supplementary Figure S4. Images were acquired using a confocal laser scanning microscope (scale bar 20 µm, magnification 63×); (**B**) Relative expression of γH2AX. * indicates statistically significant differences between samples incubated with the compound compared with control cells (*p* < 0.05); + indicates statistically significant differences between samples incubated with PARPi alone and the combination treatments (PARPi:ATRi; PARPi:CHK1i) (*p* < 0.05); (**C**) Representative Western blot images.

In situ confocal laser scanning immunofluorescence (Figure 4A and Figure 5A and Supplementary Figures S3 and S4) were consistent with the Western blot data for cleaved caspase-3 and γH2AX expression.

### 3.3. Monotherapy with ATRi/CHK1i and Combination Treatment with PARPi Activates DNA Damage Response Signaling through Chromatin Condensation and Progression through the G2/M Checkpoint

**Figure 6:**
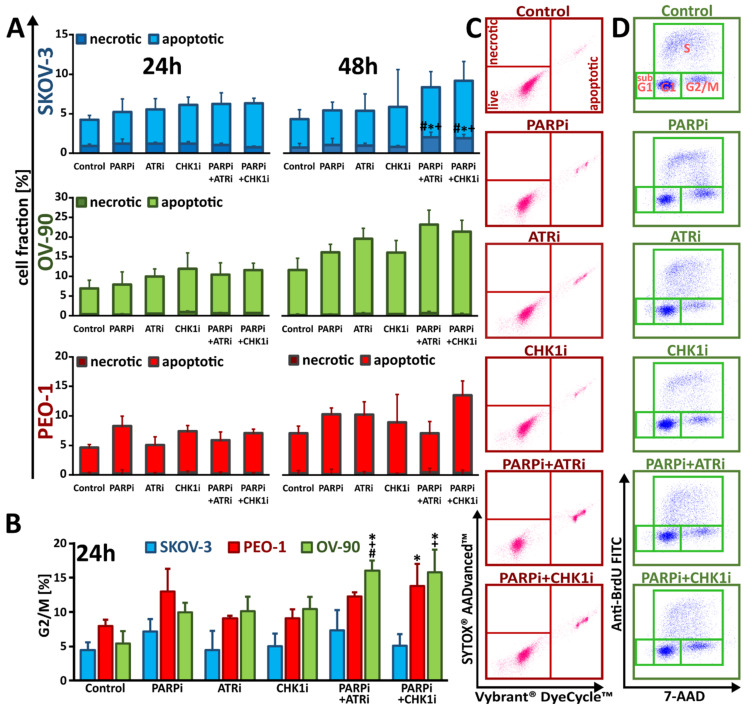
Treatment with PARPi in combination with ATR/CHK1 pathway inhibitors bypasses the cell cycle checkpoint due to an early signal of cell death. (**A**) Percentage of apoptotic and necrotic SKOV-3, PEO-1 and OV-90 cells after 24 and 48 h treatment with PARPi (4 µM), ATRi (4 µM) or CHK1i (4 µM) alone or in combination; (**B**) G2/M phase distribution of PEO-1 treated for 24 h. Data are presented as mean ± SD of 3 independent experiments. * *p* < 0.05 vs. control cells. * indicates statistically significant differences between samples incubated with the compound compared with control cells (*p* < 0.05); + indicates statistically significant differences between samples incubated with PARPi alone and the combination treatments (PARPi:ATRi; PARPi:CHK1i) (*p* < 0.05); # denotes statistically significant differences between samples incubated with ATRi or CHKi alone and their corresponding combination treatments (PARPi:ATRi; PARPi:CHK1i) (*p* < 0.05); (**C**) Representative dot plots showing induction of apoptosis and necrosis in PEO-1 cells after 48 h treatment with PARPi (4 µM), ATRi (4 µM) or CHK1i (4 µM) alone and in combination. Data for SKOV-3 and OV-90 cells are shown in Supplementary Figure S6. Individual samples are presented as data points. The population of apoptotic cells was calculated according to the presented gating strategy; (**D**) Representative dot plots demonstrating the distribution of cell cycle phases in PEO-1 cells after 24 h treatment alone and in combination. Representative dot plots for SKOV-3 and OV-90 cells are shown in Supplementary Figure S7. Individual samples are presented as data points. The population of cells in each phase of the cell cycle was calculated according to the presented gating strategy.

In contrast to SKOV-3 and PEO-1 cells, OV-90 cells were slightly more susceptible to chromatin condensation after exposure to the drugs. In SKOV-3 cells, changes in the apoptotic cellular fraction did not exceed 7%, regardless of whether the cells were exposed to monotherapy or co-treatment. Similarly, in the *BRCA^MUT^* cell line (PEO-1), the increase in the apoptotic cellular fraction after PARPi:CHK1i co-treatment was negligible (~13%) compared to olaparib monotherapy (~10%) (Figure 6A,C and Supplementary Figure S6).

The largest changes were observed in OV-90 cells, where simultaneous treatment with ATRi enhanced the effect of olaparib on the apoptotic cellular fraction from ~16% to ~23%. Similar results were found after co-treatment with CHK1i and PARPi, where an increase in the frequency of apoptosis to ~21% was observed. It should also be noted that after 48 h of treatment, only OV-90 cells were found to have significantly increased apoptosis compared to the 24 h time point. In summary, our data suggest that biochemical changes were associated with apoptotic cell death and not necrosis.

We previously confirmed that PARPi treatment activated the ATR/CHK1 pathway [10], which in turn affected cell cycle progression in the context of PARPi-induced DNA damage (Figure 6B,D and Supplementary Figure S7). Thus, we evaluated the effect of PARPi, ATRi, and CHK1i alone or in combination on cell cycle phase distribution, paying particular attention to G2/M, since cells with damaged DNA are arrested at the G2/M checkpoint prior to mitosis. We did not observe a significant change in the distribution of the G2/M phase of the cell cycle after treatment with PARPi in combination with ATRi or CHK1i in both SKOV-3 and PEO-1 cells. Furthermore, we found that PARPi treatment alone increased the frequency of G2/M cells to only 7% in SKOV-3 cells and 15% in PEO-1 cells. In both cell lines, ATRi and CHK1i alone did not increase the frequency of cells in G2/M compared to PARPi alone, with levels similar to those observed in untreated cells.

Interestingly, only OV-90 cells were more sensitive to treatment with a combination of drugs compared to monotherapy. ATRi and CHK1i administered with olaparib significantly induced G2/M arrest. Simultaneous treatment with ATRi enhanced the effect of olaparib alone (~10%) and significantly increased the frequency of G2/M cells (~16%). A similar effect, resulting in an ~15% increase in G2/M arrest, was observed when olaparib was co-administered with the CHK1 inhibitor. Cells treated with PARPi in combination with ATRi/CHK1i-induced DNA damage. Inhibition of the ATR–CHK1 pathway resulted in some cells passing through the G2/M checkpoint, leading to chromosomal damage, genomic instability, and cell death.

### 3.4. Treatment with ATRi/CHK1i Induces Aberrant Cell Cycle Progression and Increases RS Levels

**Figure 7:**
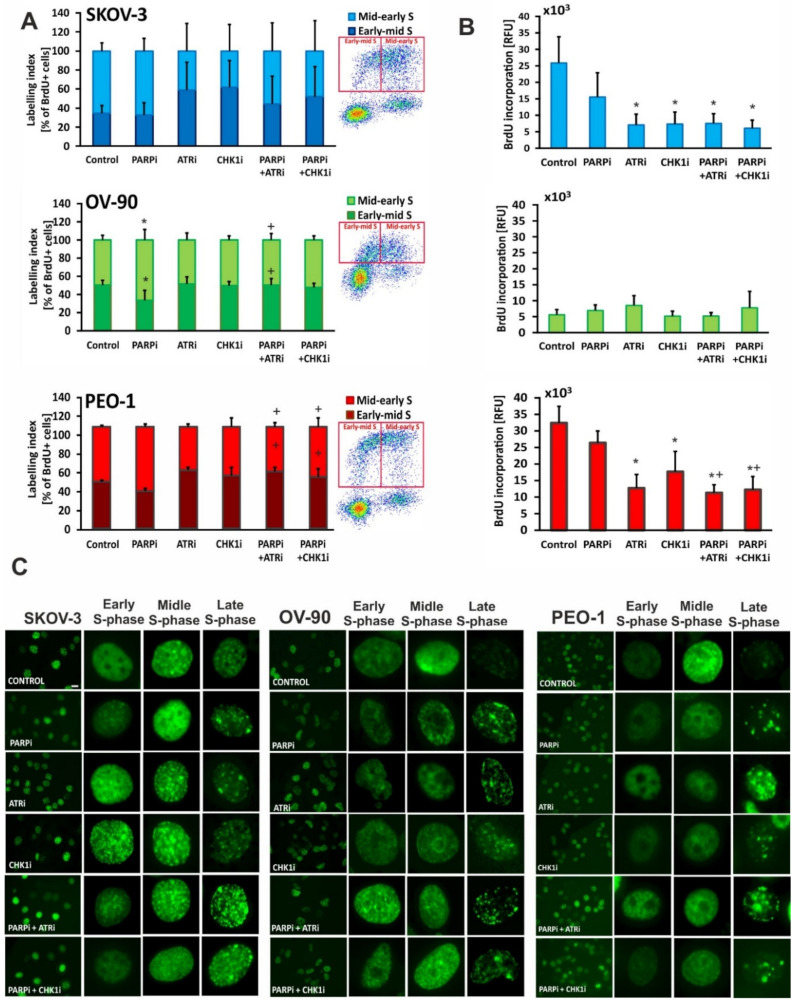
The effects of PARPi, ATRi or CHK1i alone and in combination on the S-phase of the cell cycle. (**A**) BrdU-positive heterochromatin labelling (labelling index) was used to examine the S-phase of the cell cycle after 24 h treatment with PARPi (4 µM), ATRi (4 µM) or CHK1i (4 µM) alone and in combination. The cells were gated into early mid or mid-late S-phase populations based on DNA content; (**B**) Incorporation of BrdU. Data are presented as mean ± SD of 3 independent experiments. * indicates statistically significant differences between samples incubated with the compound compared with control cells (*p* < 0.05), + indicates statistically significant differences between samples incubated with PARPi alone and the combination treatments (PARPi:ATRi; PARPi:CHK1i) (*p* < 0.05); (**C**) Fluorescence images of EdU incorporation. Based on differences in the pattern of DNA replication (EdU), cells were identified as progressing through early, mid, and late stages of the S-phase (magnification 40×, scale bar 20 µm). Enlarged fragments of photos are presented in the columns on the right side of each individual cell line panel.

To investigate the effects of kinase inhibitors in combination with olaparib on cell proliferation, we used BrdU and EdU incorporation to monitor S-phase progression. BrdU-positive cells were gated into early mid or mid-late S-phase populations. An increase in the abundance of early S-phase cells was observed in PEO-1 cells (Figure 7A), indicating the slower progression of cells through the S-phase, a sign of RS. S-phase extension was also observed in OV-90 cells after PARPi:ATRi treatment, although these changes were not statistically significant. Interestingly, treatment with ATRi and CHK1i alone or in combination with olaparib decreased BrdU intensity in S-phase, consistent with slower DNA synthesis and higher RS in OV-90 and PEO-1 cells (Figure 7B). BrdU incorporation into S-phase PEO-1 cells was more diminished after combination treatment than after treatment with olaparib alone. The combination treatment did not significantly change cell proliferation compared to ATRi or CHK1i monotherapy.

Next, we used EdU incorporation to examine the effects of the inhibitors on the three distinct S-phase stages [25,26,27]. In control cells, we observed characteristic labeling of normal S-phase: low homogeneous labeling, typical of the early S-phase (with uniform fluorescence throughout the nucleus); strong homogeneous labeling, specific to middle S-phase, and heterogeneous labeling (Figure 7C) [28]. Thus, ATR and CHK1 are critical in the early S-phase to limit replication origin firing and to suppress the formation of ssDNA.

### 3.5. Treatment with ATRi/CHK1i in Combination with PARPi Increases Chromosomal Abnormalities

**Figure 8:**
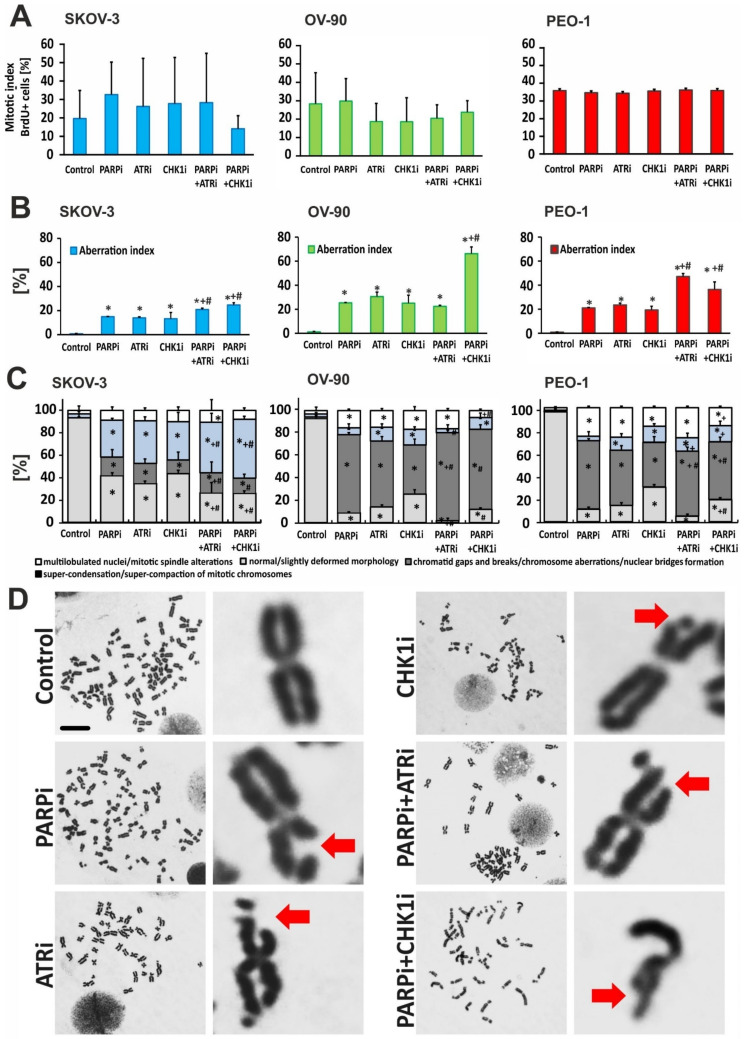
PARPi treatment in combination with CHKi and ATRi causes chromosome aberrations. DNA damage effects were measured by metaphase chromosome spread. (**A**) The mitotic index was calculated as the percent ratio between the number of dividing cells and the entire cell population. The quantification was determined by BrdU incorporation; (**B**) Aberration index (M-phase aberrant cells) was calculated as the percent ratio between the number of cells showing chromosome aberrations and all mitotic cells; (**C**) Nuclear phenotypes in PEO-1/OV-90/SKOV-3 cells were determined by Giemsa and by counterstaining with DAPI (0.1 mg/mL). Types of chromosomal aberrations were counted under the microscope (Zeiss, Jena, Germany). * indicates statistically significant differences between samples incubated with the compound compared with control cells (*p* < 0.05); + indicates statistically significant differences between samples incubated with PARPi and the corresponding combination treatments (PARPi:ATRi; PARPi:CHK1i) (*p* < 0.05); # denotes statistically significant differences between samples incubated with ATRi or CHKi and their corresponding combination treatments (PARPi:ATRi; PARPi:CHK1i) (*p* < 0.05); (**D**) Replication stress inhibitors synergize with PARPi and cause chromosomal aberrations in PEO-1 cells. Representative images of OV-90 and SKOV-3 cells are shown in Supplementary Figure S8. Red arrows show damaged chromosomes. Stained slides were analyzed using a 100× objective (scale bar 20 µm) and a Nikon ECLIPSE E600W microscope (Nikon, Warsaw, Poland).

Combination treatment using olaparib with ATRi or CHK1i can lead to a temporary latency in DNA damage, since inhibition of these kinases allows cells to pass through the G2/M checkpoint. Flow cytometry analysis of the cell cycle showed a slight decrease in the mitotic index after PARPi:CHK1i treatment in SKOV-3 cells (Figure 8A). A decrease in mitotic levels was also observed in OV-90 cells following treatment with ATRi or CHK1i, either alone or in combination with olaparib. Treatment with these inhibitors did not significantly affect the mitotic index of the PEO-1 cell line.

Despite slight changes in the mitotic index, premature initiation of mitosis follows an aberration course, because the nonreplicated regions of the genome are expressed in the form of losses or breaks in the chromosomes. Aberrant M-phase cells (included in the aberration index) showed chromosomal abnormalities. In the SKOV-3 and PEO-1 cell lines, a combination of PARPi:CHK1i and PARPi:ATRi treatment led to an increase in chromosomal aberrations compared to monotherapy (Figure 8B). However, the level of damage was 2-fold higher in the PEO-1 *BRCA^MUT^* cell line than in the SKOV-3 *BRCA^WT^* cell line.

Next, we distinguished between the specific types of chromosomal aberrations (Figure 8C). The frequency of normal cells decreased significantly after treatment with PARPi to 11.1% in PEO-1 (*BRCA^MUT^*) cells and 8.7% in OV-90 (p53^MUT^) cells compared to controls. Similarly, after ATRi treatment, the frequency of normal cells was in 14.4% in the PEO-1 cell line and 14.2% in the OV-90 cell line compared to the controls. Less significant changes were observed after treatment with CHK1i alone (~30%). Virtually no normal cells were observed after treatment with the PARPi:ATRi combination (Figure 8D). Although the PARPi:CHK1i combination had a weaker effect than PARPi:ATRi (PEO-1, 19.4% normal cells; OV-90, 12.1% normal cells), this effect was still larger than that observed after treatment with CHK1i alone. The drugs had virtually no effect on the SKOV-3 line at each dose tested. In contrast, in the OV-90 cell line, both combinations led to an increase in chromosomal aberrations (chromatid gaps and breaks, nuclear bridge formation) compared to monotherapy. Furthermore, although an increased number of aberrations was observed in the PEO-1 cell line, no differences between the combination treatment and monotherapy were found. With respect to the subcategory comprising multilobulated nuclei and mitotic spindle alterations, the greatest changes occurred in the PEO-1 cell line (26%), followed by the OV-90 (16%) and SKOV-3 (8–10%) cell lines. Noted gaps and breaks or abnormal morphology means that unrepaired DSBs enter the M-phase. Thus, blocking checkpoints with ATR or CHK1 inhibitors results in genomic instability and may lead to mitotic catastrophe (MC). Here, we found that PARPi:ATRi-treated cells that progressed into mitosis after a transient cell cycle arrest failed to separate, resulting in MC.

### 3.6. ATR Inhibition Intensifies PARPi-Induced Formation of MN in BRCA^MUT^ Cells

**Figure 9:**
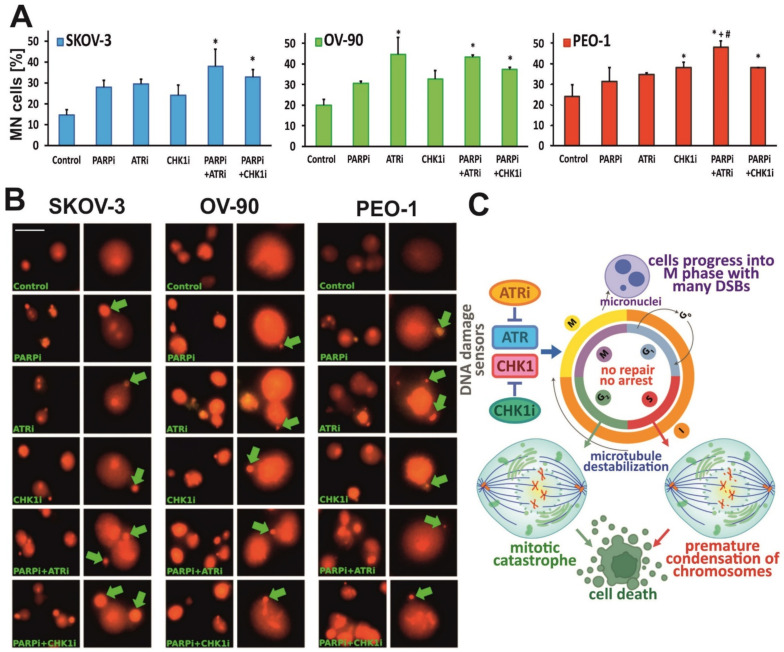
PARPi treatment in combination with ATRi/CHK1i elevates the number of cells with MN. (**A**) Number of cells with MN in OV-90, SKOV-3 and PEO-1 cell lines after 24 h treatment with PARPi (4 µM), ATRi (4 µM) or CHK1i (4 µM) alone and in combination. * indicates statistically significant differences between samples incubated with the compound compared with control cells (*p* < 0.05); + indicates statistically significant differences between samples incubated with PARPi alone and the combination treatments (PARPi:ATRi; PARPi:CHK1i) (*p* < 0.05); # denotes statistically significant differences between samples incubated with ATRi or CHKi and their corresponding combination treatments (PARPi:ATRi; PARPi:CHK1i) (*p* < 0.05); (**B**) Morphological changes after 24 h treatment with compounds related to the formation of MN. Extranuclear bodies of the damaged part of chromosome are marked by green arrows. Enlarged fragments of photos are presented in the columns on the right side of each individual cell line panel. The cells were stained with acridine orange and visualized by fluorescence microscopy (Olympus IX70, Japan; scale bar 50 µm, magnification 400×); (**C**) Ovarian cancer cells with ATR/CHK1 deficiencies progress to M phase without G2/M checkpoint arrest. ATRi/CHK1i induces premature chromosome condensation (PCC) by bypassing the internal S-phase checkpoint mechanism. The failure of G2/M checkpoint arrest causes severe DNA fragmentation such as unrepaired double-strand breaks (DSBs) during M phase, which result in multiple MN. Cells with multiple nuclear fragmentations undergoing MC.

MN are pieces of chromosomes or entire chromosomes that are formed during cell division. MN are not incorporated into one of the two main daughter nuclei [29]. After the telophase, they form a rounded body inside the cytoplasm, which is separate from the main nuclei [30]. Here, we found that a mitosis-dependent type of MN formation, identified as a typical consequence of lost or lagging chromosomal fragments during the anaphase–telophase transition (Figure 9). Following 24 h treatment, ATRi induced a statistically significant increase in the frequency of cells with MN (44.7% vs. control) in OV-90 cells. Combination treatment with PARPi and ATRi, as well as PARPi and CHK1i, induced similar frequencies of MN (43.3% and 37.4%, respectively). In the SKOV-3 and OV-90 lines, no beneficial effect of the combination was observed. Monotherapy with either PARPi or CHK1i did not significantly affect the number of cells with MN. However, in SKOV-2 cells, elevated levels of MN were only detected after treatment with a combination of PARPi and ATRi (38%) or PARPi and CHK1i (32.7%). In PEO-1 cells, ATRi synergized with PARPi to increase the frequency of MN to 48% in contrast to treatment with PARPi or ATRi alone.

## 4. Discussion

Ovarian cancer remains a highly lethal disease [31]. PARP inhibitors are now used as a maintenance therapy for patients with *BRCA^MUT^* epithelial ovarian cancer and as a treatment of relapsed *BRCA^MUT^* ovarian cancer in patients who have been treated with two or more chemotherapies before [32,33]. Despite their effectiveness, resistance to PARP inhibitors has been clinically reported [34,35,36]. Thus, it is critical to determine the mechanism of action of PARPi, as well as other inhibitors that compromise genomic stability linked to cell cycle disruption. There is a strong connection between genomic instability acquired during the S-phase and chromosomal instability following mitotic progression. The purpose of our study was to examine the short-term (up to 48 h) efficacy of PARPi together with DDR-targeting agents. We investigated whether premature mitotic entry induced by ATR/CHK1 inhibition increases genomic instability in *BRCA^MUT^* and *BRCA^WT^* HGSOC. We demonstrated that a combination of inhibitors was not more cytotoxic than monotherapy. However, despite the weak cytostatic activity of PARP, treatment with CHK1i or ATRi in combination with PARPi results in an increase in replication disorders due to a lack of checkpoint control by CHK1/ATR and PARP. ATR and CHK1 kinase inhibitors disrupt the cell cycle and as a result, cells with high levels of DSBs enter mitosis prematurely in the presence of unrepaired DNA. The combination of olaparib with inhibitors of the ATR/CHK1 pathway generated chromosomal abnormalities, independent of the *BRCA^MUT^* status of the cells, and formed micronuclei. Our data suggest that DNA DSB lesions do not immediately lead to cell death, but can progress into mitosis, resulting in chromatin aberrations. Chromosome instability might cause cell death by MC. Abrogation of the ATR-initiated checkpoint cascade mediated through CHK1 has previously been shown to direct cells into MC [37].

DDR is controlled not only by the concentration and persistence of DNA lesions, but also by the proliferative status of the damaged cell. Previously, we examined the effect of prolonged PARPi, ATRi and CHK1i treatment and confirmed a differential drug response profile, which was dependent on *BRCA* status [10]. In this study, abnormalities in the proliferation process were initially observed 24 h after combined treatment with PARPi and ATRi, with a significantly stronger effect seen after 48 h. Changes in the proliferation machinery were manifested as a decrease in the mitotic index and an increase in heterochromatin formation. Our studies showed that the combination of ATRi/CHK1i with PARPi is no more cytotoxic than monotherapy with ATRi and CHK1. This may indicate that at the selected time and dose, olaparib in the tested lines has a cytostatic effect. Combining olaparib with ATRi was cytotoxic in ATM-deficient cells and cytostatic in ATM^WT^ cells [38]. Cytotoxic and cytostatic effects of olaparib and IR were induced in a PTEN (phosphatase and tensin homolog) independent manner in the endometrial HEC-6 cells [39]. Cell survival variance of triple-negative breast cancers were impacted at 40% by the time of olaparib exposure, at 39% by radiotherapy, at 14% by the olaparib concentration, and at 9% by the type of cell line [40]. In our study, the cytostatic effect of olaparib may require prolonged dosing for therapeutic effect [41]. Although the PEO-1 *BRCA^MUT^* cell line was the most sensitive to treatment with RS kinase inhibitors, we found that the ATRi and CHK1i were cytotoxic and genotoxic to *BRCA^WT^* cells. Loss of the integral HR pathway components in cancers with non-*BRCA* HR defects due to germline or somatic mutations in *PALB2*, *RAD51B*, *RAD51C*, or *RAD51D*, or their inactivation through promoter methylation, might be the cause of olaparib susceptibility [42].

Next, we examined chromatin condensation as an early marker of repaired DNA damage after treatment with the tested inhibitors. Our data showed slight changes in chromatin condensation in all cell lines. Significant differences were observed in the SKOV-3 line after treatment with both drug combinations. Previous studies have demonstrated that chromatin needs to be dynamically reorganized to properly orchestrate DDR [43]. Moreover, chromatin compaction may provide the structural and molecular environment to mimic the DDR amplification step, and therefore trigger ATM and ATR signaling independent of the DNA lesions [43,44]. PARP1 selectively binds to transcriptionally active chromatin and controls the fidelity of gene transcription [45,46]. Inactive forms of PARP1 may contribute to the formation of condensed chromatin structures [47]. On the other hand, histone eviction and local chromatin decondensation have been observed in the presence of DSBs, which is then followed by chromatin compaction [48] and acceleration of DDR downstream pathways, including signaling through the CHK1 and CHK2 kinases [43,49].

ATR and CHK1 inhibitors result in the accumulation of DNA damage in mitosis, including MN formation and thus cell death. The beneficial effect of the PARPi:ATRi combination on MN was seen only in the PEO-1 *BRCA^MUT^* cell line. Lagging chromosomes and MN are elevated upon PARP1/2 depletion or inhibition, following the accumulation of unrepaired or inappropriately repaired DNA lesions during the S-phase that progress into mitosis [14]. Olaparib was shown to induce MN formation and anaphase chromatin bridges, which are hallmarks of chromosome missegregation [50]. ATRi was found to promote the generation of acentric dysfunctional MN and abrogate radiation-induced G2 cell-cycle checkpoint arrest due to aberrant mitosis and MC [51]. ATRi extended mitotic duration, associated with lagging chromosomes, anaphase bridges and MN formation upon completion of mitosis. Combined PARPi and ATRi treatment resulted in severely extended mitotic duration, eventually leading to genome disintegration and catastrophic damage [52]. Treatment with PARPi and ATRi in combination were also found to be effective in ATM-deficient tumors, elevating the number of MN to a greater extent than olaparib monotherapy [38]. Our findings are partly consistent with a study by Schoonen et al., where increased mitotic defects and elevated numbers of MN in *BRCA2*-defective cells were observed after PARP inhibition, while the number of MN increased upon ATR inhibition [53].

One of the consequences associated with the presence of MN is cell death. In DDR, MC is induced when chromatin condensation and spindle assembly occur on incompletely replicated DNA to cause chromosome fragmentation [54]. PARP plays a crucial role in the maintenance of chromosomal stability to avoid an increase in cells with structural chromosomal aberrations. A higher frequency of chromatid breaks, anaphase bridges and MN have been found following PARP1 inhibition, particularly in response to genotoxic stress [55]. Another study confirmed that olaparib-treated cells might die by MC [14]. In the unperturbed S-phase, ATR is activated at a low level during the early S-phase, and its activity declines at the end of the S-phase. ATR inhibition leads to the premature accumulation of cyclin B through the expression of forkhead box protein M1 (FOXM1) in S-phase cells [56]. We demonstrate that ATRi and CHK1i alone impact replication throughout the S-phase as a result of raising the RS. Previously, we reported that the activation of the S-phase checkpoint in response to DNA damage might be associated with CHK1 phosphorylation in PARPi-treated cells [10]. It is known that the basal levels of CHK1 protein during the S-phase inhibit mitotic entry. Inhibiting CHK1 has the opposite effect and has been found to increase the initiation of DNA synthesis, as well as DNA damage [57]. However, despite olaparib-induced checkpoint activation, cancer cells still progress into mitosis. Extensive DNA damage in mitosis causes metaphase arrest due to defective kinetochore attachment and activation of the spindle-assembly checkpoint (SAC) [58].

Metaphase arrest due to DNA damage leads to MC, whereby cells die by apoptosis or by avoiding mitotic arrest [59]. Here, apoptosis was found to be significantly increased with the ATRi/CHK1i monotherapy in both *BRCA^MUT^* and HR-proficient cell models. ATRi and CHK1i-treated cells that underwent apoptosis had DNA content consistent with the early S-phase, suggesting that cells carrying DNA lesions into the S-phase, in the absence of functional ATR, are predisposed to higher levels of RS and intensified cell death [60]. Treatment with olaparib alone was unable to increase the expression of cleaved caspase-3, an apoptosis marker, in the SKOV-3 cell line [61]. Here, the apoptotic cellular fractions assessed by double staining with Hoechst 33258/PI and by the externalization of phosphatidylserine did not significantly increase after olaparib monotherapy, similar to changes in caspase-3 levels, suggesting that olaparib alone was insufficient to induce cell death by apoptosis in the tested time range. ATRi and CHK1i monotherapies were more efficient in apoptosis induction, elevating cleaved caspase-3 levels and the frequency of apoptotic cells in the OV-90 and *BRCA^MUT^* PEO-1 cell lines. Previously, we assessed the overall level of DNA damage, including SSBs and DSBs, using the alkaline and neutral variants of the comet assay, and found that ATRi and CHK1i had a marked effect on DNA strand breaks [10]. In the current study, DSBs were monitored using the DSB marker, γH2AX. PIKK-mediated phosphorylation of histone H2AX generates γH2AX, which functions as a chromatin marker for the assembly of repair and signaling complexes [54]. Chromatin regulatory mechanisms are engaged during DDR to promote lesion recognition, repair, and signal transduction [54]. Unresolved replication lesions do not necessarily block mitotic entry, but progress into mitosis, leading to chromosome instability. In our study, weak expression of γH2AX was observed after PARPi treatment in all cell lines tested. Our findings are similar to a study by Fleury et al., which demonstrated that olaparib treatment alone was not effective enough to increase the number of γH2AX foci and even MN in HGSOC cell lines [62]. Here, we confirmed the presence of γH2AX after the ATRi monotherapy, PARPi:ATRi and PARPi:CHK1i combined therapy in PEO-1 cells. Our findings are consistent with previous studies [53,63]. Furthermore, consistent with a study by Huntoon et al., we found that γH2AX levels were increased after ATRi and CHK1i treatment alone or in combination with PARPi in *BRCA^MUT^* and *BRCA^WT^* cells [64]. Interestingly, we observed high γH2AX levels in the SKOV-3 (*BRCA^WT^*) line. However, when comparing the control values of the tested cell lines, it is worth noting that the SKOV-3 cells had lower levels of γH2AX than PEO-1 cells. Our data are consistent with other studies. Phosphorylated histone H2AX was observed following PARPi treatment to the same extent in wild-type and *BRCA1/2*-deficient cell lines, suggesting that DSBs occur independent of *BRCA* function [65].

Inhibition of PARP1/2 during mitosis does not lead to the same mitotic phenotypes that arise in response to PARP1/2 inhibition during DNA replication in the S-phase. Furthermore, premature loss of cohesion occurred when olaparib was added during the S-phase (S-phase stalling), suggesting that replication fork blockage due to PARP entrapment leads to a loss of cohesion and subsequent defects in mitosis. Olaparib causes loss of sister chromatid cohesion. Loss of cohesion in interphase cells causes chromatid scattering in metaphase cells, metaphase arrest and cell death [66]. It has been shown that PARP trapping during the S-phase is required for the induction of mitotic chromosomal bridges [14]. Chromosomal aberrations in either PARPi:ATRi- or PARPi:CHK1i-treated cells imply inappropriate repair of DSBs before entry into mitosis, increased replication fork collapse and abrogation of the G2/M-phase checkpoints in PEO-1 and SKOV-3 cells. Only in OV-90 cells were ATRi or CHK1i found to enhance the olaparib effect and promote a small increase in G2/M cells (~16%). Inhibition of ATR or CHK1 led to an increase in olaparib-induced markers of RS [67]. Other studies have confirmed that a lack of G2/M activation and/or spindle checkpoints in cells suffering from DNA damage occurs due to inhibition of the ATR/CHK1 pathway [54,68].

## 5. Conclusions

MC is not a separate mode of cell death, but rather a process preceding cell death, which can occur through necrosis or apoptosis in ovarian cancer cells [37]. Importantly, PARPi in short incubation times show cytostatic effect rather than cytotoxic activity. Therefore, the combination of ATRi/CHK1i with PARPi is not more cytotoxic than monotherapy. Our research indicates that the cytotoxic effect of the combination is time dependent. The final outcome of cell death depends on the molecular profile of the cell and, in our study, is independent on *BRCA* status. There is undoubtedly a connection between ATR/CHK1 inhibition and DNA damage-induced cell-cycle disruption [69]. Both ATRi and CHK1i tested in this study were found to cause inappropriate entry into mitosis, leading to chromosomal aberrations, genome instability, and apoptosis. We confirmed that ATRi and CHK1i cytotoxicity was due to mitotic dysfunction. Additional research in vivo is required to further explore the effect of PARPi:ATRi and PARPi:CHK1i on genome stability and cancer cell survival, which may also depend on the genetics of the tumor.

## Data Availability

The datasets presented during the current study are available from the corresponding author on reasonable request.

## Supplementary Materials

The following supporting information can be downloaded at: https://www.mdpi.com/article/10.3390/cells11121889/s1, Figure S1: ATRi/CHK1i monotherapy and combination treatment caused cell death. Fluorescence images of the apoptotic and necrotic changes caused by treatment with the compounds in SKOV-3 and OV-90 cells. Representative cells are marked with a dashed line and enlarged. The cells were divided into four categories as follows: live cells (dark blue fluorescence), early apoptotic (bright blue fluorescence), late-apoptotic (pink–violet fluorescence), and necrotic cells (red fluorescence). Apoptotic and necrotic changes were visualized after double staining with Hoechst 33258/PI under a fluorescence microscope (Olympus IX70; scale bar 50 µm; magnification 400×); Figure S2: ATRi/CHK1i monotherapy and combination treatment caused phosphatidylserine externalization. Representative dot plots showing induction of apoptosis and dead SKOV-3 and OV-90 cells after 48 h treatment with PARPi (4 µM), ATRi (4 µM) or CHK1i (4 µM) alone and in combination. Individual samples are presented as data points. The population of apoptotic cells was calculated according to the presented gating strategy; Figure S3: ATRi/CHK1i increases caspase-3 expression. For immunofluorescence staining, SKOV-3 and OV-90 cells were stimulated with PARPi, ATRi or CHK1i alone, or the combination of PARPi:ATRi or PARPi:CHK1i at 4 µM and labeled with antibodies against caspase-3 (red colour). Images were acquired using a confocal laser scanning microscope (scale bar 20 µm, magnification 63×); Figure S4: ATRi/CHK1i increases γH2AX expression. For immunofluorescence staining, SKOV-3 and OV-90 cells were stimulated with PARPi, ATRi or CHK1i alone, or the combination of PARPi:ATRi or PARPi:CHK1i at 4 µM and labeled with antibodies against γH2AX (green colour). Images were acquired using a confocal laser scanning microscope (scale bar 20 µm, magnification 63×); Figure S5: γH2AX expression in SKOV-3, OV-90 and PEO-1 control cells presented as arbitrary area value; Figure S6: Representative dot plots showing induction of apoptosis and necrosis in SKOV-3 and OV-90 cells after treatment with PARPi (4 µM), ATRi (4 µM) or CHK1i (4 µM) alone and in combination. Individual samples are presented as data points. Population of apoptotic [Q2 (Vybrant-positive and Sytox-positive) + Q4 (Vybrant-positive and Sytox-negative)] and necrotic cells [Q1 (Vybrant-negative and Sytox-positive)] was calculated according to the presented gating strategy; Figure S7: Representative dot plots showing the distribution of cell cycle phases in SKOV-3 and OV-90 cells after 24 h treatment with PARPi (4 µM), ATRi (4 µM) or CHK1i (4 µM) alone and in combination. Individual samples are presented as data points; Figure S8: Replication stress inhibitors synergize with PARPi to induce chromosomal aberrations in (A) OV-90 and (B) SKOV-3 cells. Red arrows show damaged chromosomes, (50 metaphase spreads in each group were counted). Stained slides were analyzed using a 100× objective and a Nikon ECLIPSE E600W microscope (Nikon, Warsaw, Poland).
