# Supplementary material for: The Influence of PARP, ATR, CHK1 Inhibitors on Premature Mitotic Entry and Genomic Instability in High-Grade Serous BRCAMUT and BRCAWT Ovarian Cancer Cells
Source: Cells. 2022 Jun 10;11(12):1889. doi: 10.3390/cells11121889 (PMC9221516; doi:10.3390/cells11121889)
Supplement: Supplementary file 1 [file cells-11-01889-s001.zip › cells-1715253-supplementary.pdf]

Supplementary files

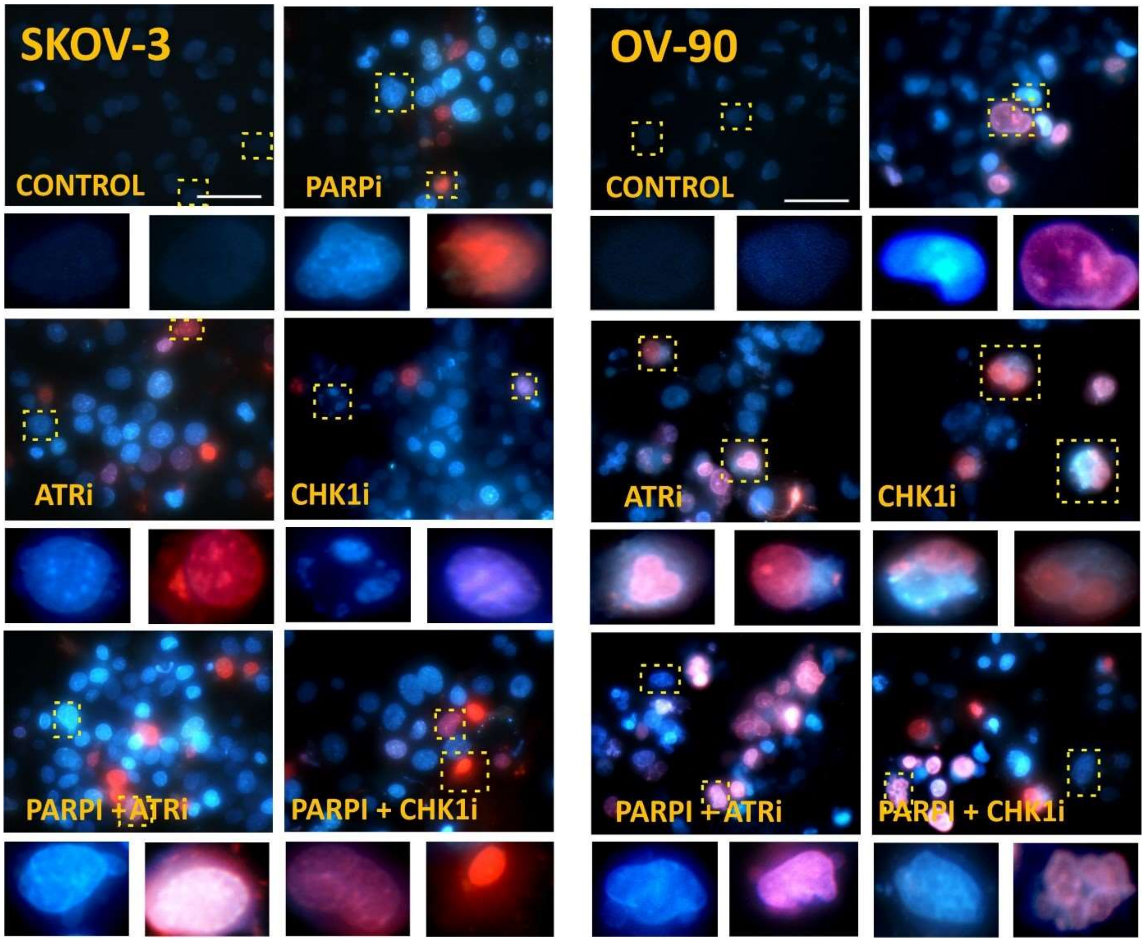

Figure S1. ATRi/CHK1i monotherapy and combination treatment caused cell death. Fluorescence images of the apoptotic and necrotic changes caused by treatment with the compounds in SKOV-3 and OV-90 cells. Representative cells are marked with a dashed line and enlarged. The cells were divided into four categories as follows: live cells (dark blue fluorescence), early-apoptotic (bright blue fluorescence), late-apoptotic (pink–violet fluorescence), and necrotic cells (red fluorescence). Apoptotic and necrotic changes were visualized after double staining with Hoechst 33258/PI under a fluorescence microscope (Olympus IX70; scale bar 50 μm; magnification 400×).

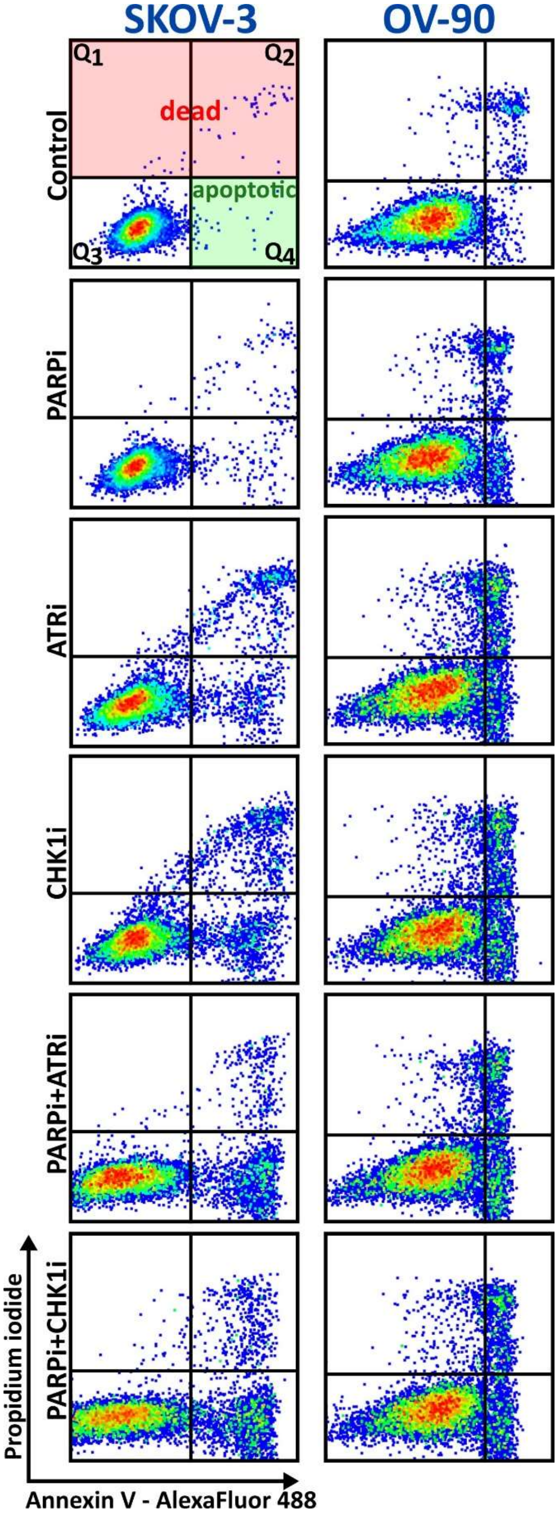

Figure S2. ATRi/CHK1i monotherapy and combination treatment caused phosphatidylserine externalization. Representative dot plots showing induction of apoptosis and dead SKOV-3 and OV-90 cells after 48h treatment with PARPi (4 μM), ATRi (4 μM) or CHK1i (4 μM) alone and in combination. Individual samples are presented as data points. The population of apoptotic cells was calculated according to the presented gating strategy.

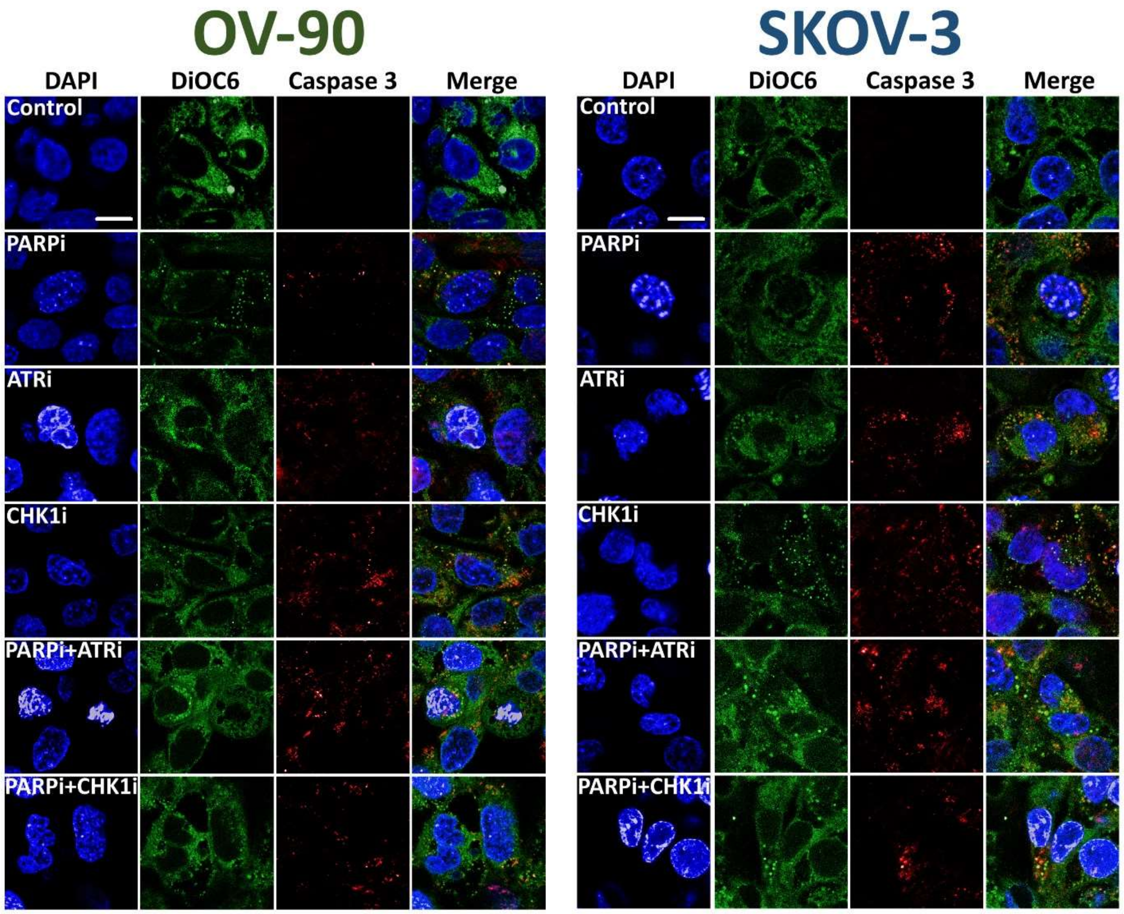

Figure S3. ATRi/CHK1i increases caspase-3 expression. For immunofluorescence staining, SKOV-3 and OV-90 cells were stimulated with PARPi, ATRi or CHK1i alone, or the combination of PARPi:ATRi or PARPi:CHK1i at 4 μM and labeled with antibodies against caspase-3 (red colour). Images were acquired using a confocal laser scanning microscope (scale bar 20 μm, magnification 63×).

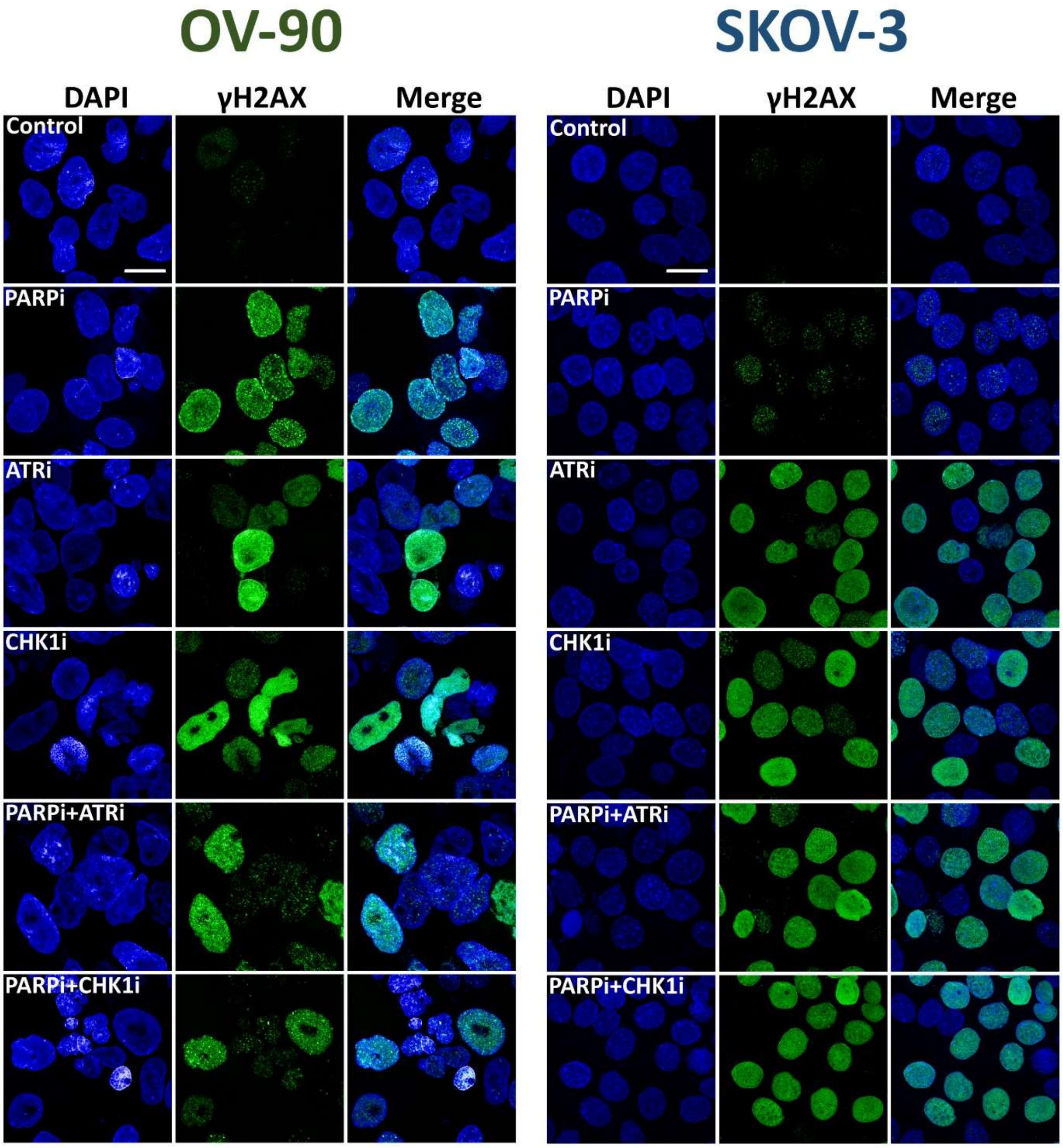

Figure S4. ATRi/CHK1i increases γH2AX expression. For immunofluorescence staining, SKOV-3 and OV-90 cells were stimulated with PARPi, ATRi or CHK1i alone, or the combination of PARPi:ATRi or PARPi:CHK1i at 4 μM and labeled with antibodies against γH2AX (green colour). Images were acquired using a confocal laser scanning microscope (scale bar 20 μm, magnification 63×).

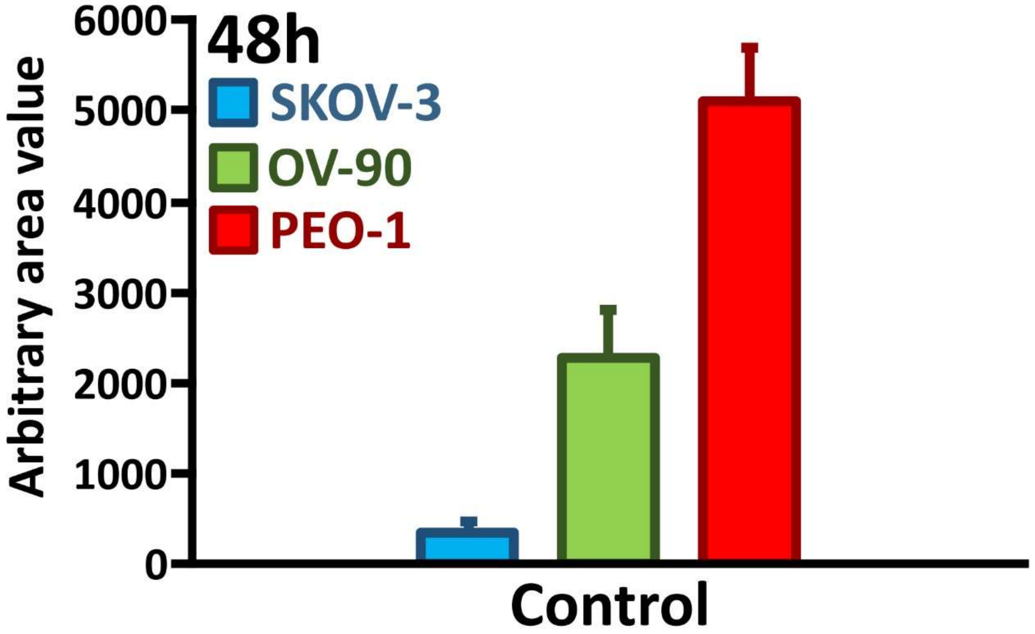

Figure S5. γH2AX expression in SKOV-3, OV-90 and PEO-1 control cells presented as arbitrary area value.

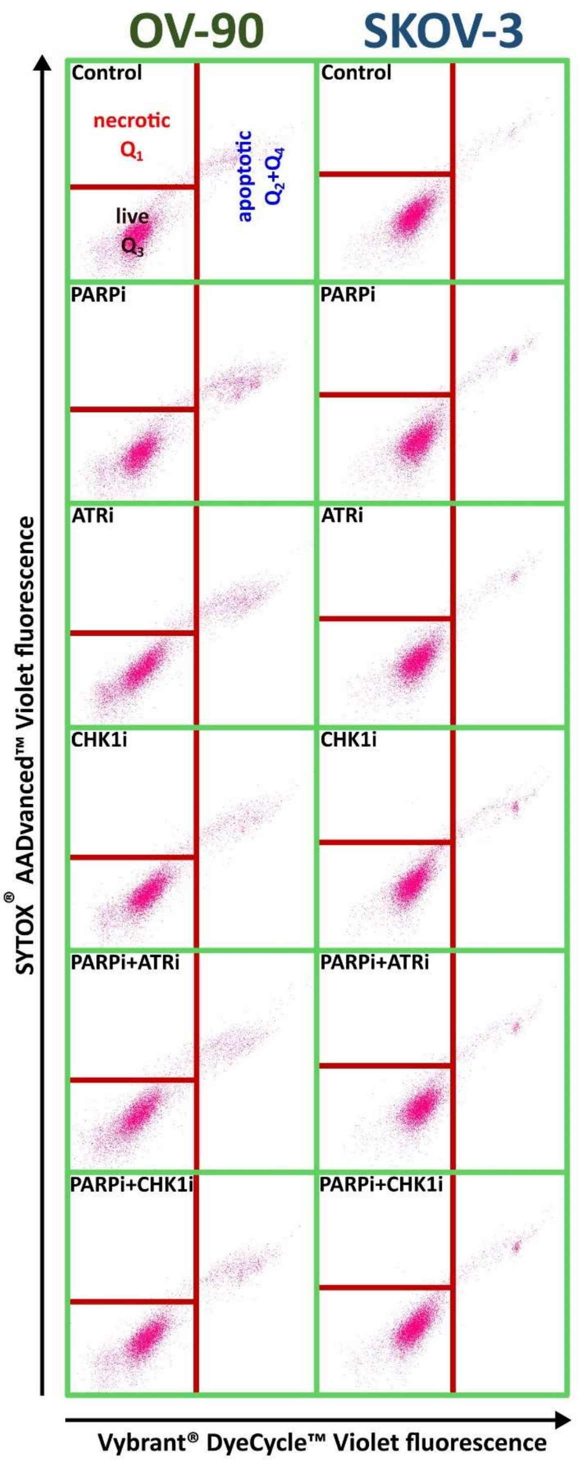

Figure S6. Representative dot plots showing induction of apoptosis and necrosis in SKOV-3 and OV-90 cells after treatment with PARPi (4 μM), ATRi (4 μM) or CHK1i (4 μM) alone and in combination. Individual samples are presented as data points. Population of apoptotic [Q2 (Vybrant-positive and Sytox-positive) + Q4 (Vybrant-positive and Sytox-negative)] and necrotic cells [Q1 (Vybrant-negative and Sytox-positive)] was calculated according to the presented gating strategy.

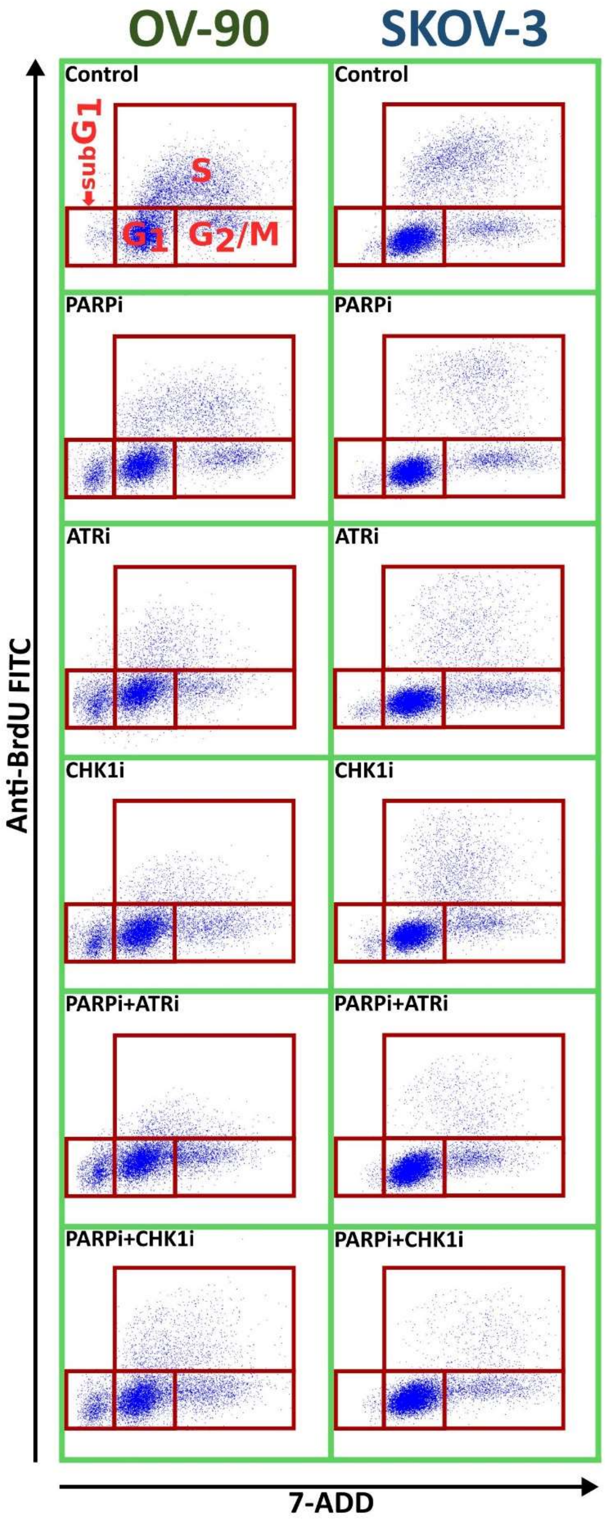

Figure S7. Representative dot plots showing the distribution of cell cycle phases in SKOV-3 and OV-90 cells after 24 h treatment with PARPi (4 μM), ATRi (4 μM) or CHK1i (4 μM) alone and in combination. Individual samples are presented as data points.

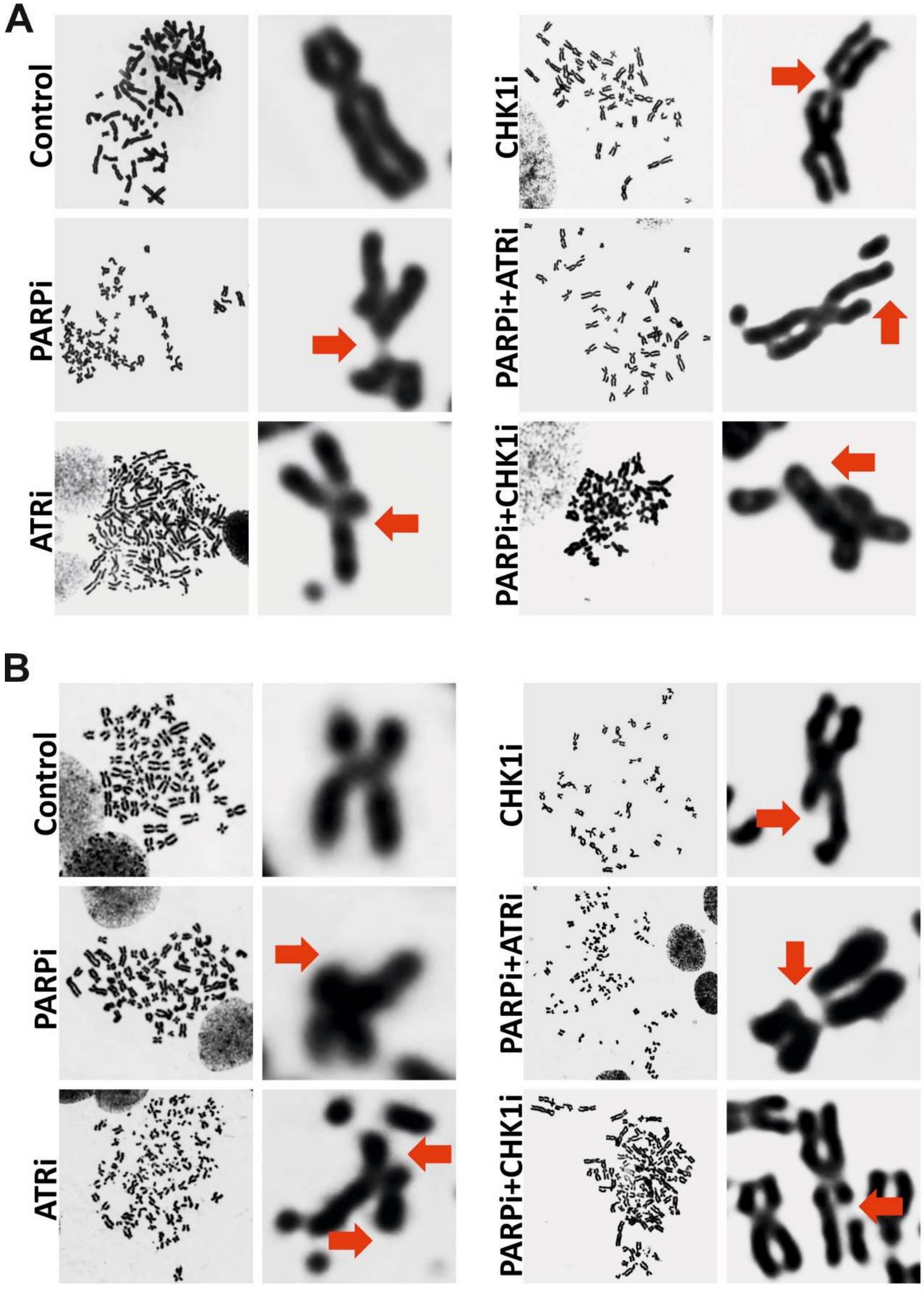

Figure S8. Replication stress inhibitors synergize with PARPi to induce chromosomal aberrations in **(A)** OV-90 and **(B)** SKOV-3 cells. Red arrows show damaged chromosomes, (50 metaphase spreads in each group were counted). Stained slides were analyzed using a 100× objective and a Nikon ECLIPSE E600W microscope (Nikon, Warsaw, Poland).
